# Impaired consolidation of spatial memory during sleep in patients with leucine-rich glioma-inactivated 1-associated limbic encephalitis

**DOI:** 10.1093/braincomms/fcag255

**Published:** 2026-07-06

**Authors:** Julius Rave, Annika Hanert, Younes Adam Tabi, Yasmin Fiedler, Sarah Philippen, Oliver Granert, Frank Leypoldt, Jan Born, Andrea Burgalossi, Robby Schönfeld, Carsten Finke, Thorsten Bartsch

**Affiliations:** Department of Neurology, Memory Disorders and Plasticity Group, University Hospital Schleswig-Holstein, University of Kiel, 24105 Kiel, Germany; Clinician Scientist Program in Evolutionary Medicine, University of Kiel, 24105 Kiel, Germany; Department of Neurology, Memory Disorders and Plasticity Group, University Hospital Schleswig-Holstein, University of Kiel, 24105 Kiel, Germany; Department of Neurology, Memory Disorders and Plasticity Group, University Hospital Schleswig-Holstein, University of Kiel, 24105 Kiel, Germany; Clinician Scientist Program in Evolutionary Medicine, University of Kiel, 24105 Kiel, Germany; Department of Neurology, Memory Disorders and Plasticity Group, University Hospital Schleswig-Holstein, University of Kiel, 24105 Kiel, Germany; Department of Neurology, Memory Disorders and Plasticity Group, University Hospital Schleswig-Holstein, University of Kiel, 24105 Kiel, Germany; Department of Neurology, Memory Disorders and Plasticity Group, University Hospital Schleswig-Holstein, University of Kiel, 24105 Kiel, Germany; Department of Neurology, Memory Disorders and Plasticity Group, University Hospital Schleswig-Holstein, University of Kiel, 24105 Kiel, Germany; Department of Neuroimmunology, Institute for Clinical Chemistry UKSH Campus Kiel and Lübeck, 24105 Kiel and 23538 Lübeck, Germany; Institute for Medical Psychology and Behavioral Neurobiology, University of Tübingen, 72076 Tübingen, Germany; Institute of Neurobiology, Werner-Reichardt Center for Integrative Neuroscience, University of Tübingen, 72076 Tübingen, Germany; Department of Psychology, Martin-Luther-Universität Halle-Wittenberg, 06108 Halle (Saale), Germany; Department of Neurology, Cognition in Neurological Disorders Group, University Hospital Charité & Berlin School of Mind and Brain, 10117 Berlin, Germany; Department of Neurology, Memory Disorders and Plasticity Group, University Hospital Schleswig-Holstein, University of Kiel, 24105 Kiel, Germany; Clinician Scientist Program in Evolutionary Medicine, University of Kiel, 24105 Kiel, Germany

**Keywords:** Memory consolidation, Sleep, Spatial navigation, Hippocampus, LGI1

## Abstract

Sleep promotes the systems consolidation of hippocampus (HC)-dependent spatial memories by reprocessing of previously encoded hippocampal representations. Hippocampal reprocessing involves pattern separation and pattern completion as central hippocampal functions performed by the dentate gyrus (DG) and cornu ammonis region 3 (CA3), respectively. The leucine-rich, glioma inactivated 1 (LGI1)-associated limbic encephalitis (LE) is an autoimmune brain disorder particularly affecting the DG and CA3 regions, thereby impairing hippocampal function. We studied 15 LGI1 patients (and matched healthy controls) to examine hippocampal contributions to the sleep-associated consolidation of spatial memory. Spatial memory was assessed using the virtual Morris water maze (VWM) during learning before nocturnal sleep. Spatial retrieval of target locations (as indicated by dwell time in target area) was tested in the next morning, with separate trials testing pattern separation and pattern completion functions, as well as place memory precision and reversal learning capabilities. Leucine-rich, glioma inactivated 1-associated limbic encephalitis (LGI1-LE) patients were able to learn and retrieve spatial locations, albeit to a lesser extent than controls. Recall of place memories was decreased in LGI1-LE patients in comparison with learning performance before sleep and with healthy controls, especially in trials assessing pattern separation. Moreover, at recall, LGI1 patients showed a less flexible adaptation to the reversal learning task, in comparison with the controls. Sleep quality, macro-sleep architecture and EEG slow oscillations (SOs) and spindles were comparable in both groups. However, in LGI1-LE patients, phase–amplitude coupling of SO–spindle events appeared diminished although the group difference did not remain significant after correction for multiple comparisons. In addition, a negative correlation between spindle density and retrieval of target locations was observed. Magnetic resonance imaging confirmed smaller volumes of the HC and its subfields (subiculum, CA1, CA3, DG) in the patients. Divergent structure–function relationships emerged between patients with LGI1-associated encephalitis and healthy controls: In patients, larger volumes of DG and CA3 were associated with weaker sleep-dependent consolidation but greater stability under cue deprivation. In controls, larger hippocampal, CA1, and subicular volumes correlated with better memory retrieval and reversal learning performance. Our results show an impaired sleep-associated consolidation of spatial memory in LGI1-LE patients highlighting the involvement of DG and CA3 areas in sleep-associated spatial memory formation and cognitive flexibility.

## Teaser

Rave *et al*. report impaired sleep-associated spatial memory consolidation in leucine-rich, glioma inactivated 1 (LGI1)-associated limbic encephalitis. Patients showed preserved learning but reduced post-sleep recall, especially regarding pattern separation, and impaired reversal learning. Despite normal sleep architecture, altered slow oscillation–spindle coupling and subfield related structure–function changes indicate disrupted hippocampal network mechanisms.

## Graphical Abstract

**Graphical Abstract:**
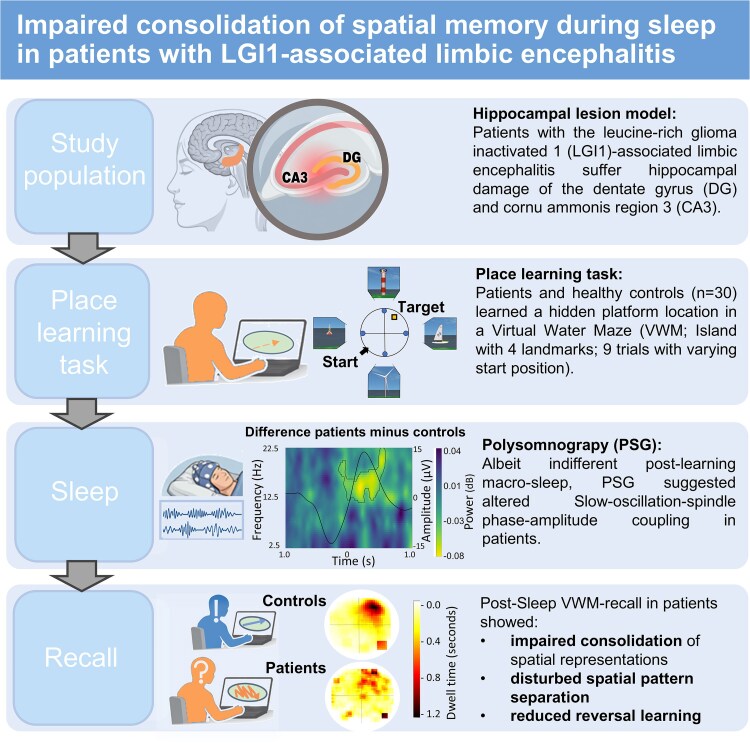

## Introduction

While the formation of robust spatial memory substantially relies on neocortical networks,^1-3^ it most critically depends on hippocampal circuits.^4-6^ Hippocampal cornu ammonis (CA1)-specific pyramidal cells and granular cells of the dentate gyrus (DG) function as place cells forming place fields in which objects and landmarks are defined in vectoral relation to each other.^4,7^ Through multi-level integration of these fields, the hippocampus (HC) constructs cognitive maps of spatial environments.^8^

Sleep plays an active role in the consolidation of spatial memory, mediating the transfer from hippocampal short-term to neocortical long-term stores.^9-11^ According to the active systems consolidation concept,^10^ newly encoded memories are transformed into long-term representations during slow-wave sleep (SWS), under top–down control of neocortical slow oscillations (SOs). The depolarizing SO up-phases promote coordinated reactivation of hippocampal memory traces via sharp-wave ripples (SWRs) and thalamocortical spindles.^12-14^ This synchronization facilitates spindle–ripple events, coupling SWRs and reactivated information to the excitable spindle down-phase.^15-17^

During the sleep-associated reprocessing of representations, CA3 and DG support distinct functions: CA3 neurons replay neuronal firing sequences of the prior wake state,^18-20^ assumed to support memory consolidation and schema formation.^21-23^ Inputs from the DG modulate CA3 activity,^24^ promoting a stronger pattern separation function.^25^

Dentate gyrus and CA3 also play complementary roles during learning.^26,27^ The DG enables discrimination of (spatially) similar but distinct inputs (pattern separation), thereby enabling successful retrieval of competing spatial environments.^28-30^ In contrast, CA3 supports the retrieval of a memory from incomplete cues (pattern completion).^29,31^

While remapping—the reorganization of spatial firing patterns in response to changing contexts—is a property of all hippocampal subfields, CA1 exhibits the most rapid and pronounced global remapping.^27^ This remapping capacity likely contributes to the HC broader role in supporting flexible behaviour through context-sensitive memory encoding.^32^ One behavioural paradigm to assess such flexibility is spatial reversal learning, which requires reassigning stimulus–reward associations when previously learned contingencies change.^33-37^ In addition to the dorsolateral striatum and prefrontal cortex, the HC supports this process by flexibly reconfiguring spatial representations.^38,39^

Crucially, this remapping flexibility depends on pattern separation and completion within DG and CA3, which are essential for distinguishing and retrieving competing memories.^40^ We previously showed in healthy participants that sleep stabilizes pattern separation performance, correlating with non-REM oscillatory markers of hippocampal reactivation.^25^

The leucine-rich, glioma inactivated 1 (LGI1)-associated limbic encephalitis (LE) provides a unique opportunity to study the role of hippocampal subfields in human memory formation.^41^ Leucine-rich, glioma inactivated 1 is a secreted glycoprotein that modulates synaptic transmission and plasticity by interacting with Kv1 channels, AMPA receptors and the Nogo receptor 1 (NgR1).^42-44^ While LGI1 is expressed throughout the brain, it is particularly enriched in granule cells of the DG and pyramidal cells of the CA3 region.^43-46^

In LGI1-LE, autoantibodies disrupt Kv1.1 and Kv1.2 channel clustering, broadening presynaptic action potentials and increasing neurotransmitter release, ultimately leading to a loss of LGI1 function and resulting in neuronal hyperexcitability and impaired neuroplasticity, particularly in DG and CA3.^44-48^

Clinically, the acute stage of LE is characterized by sleep and memory impairments, psychiatric symptoms and epileptic seizures, with disabling memory deficits often persisting into the chronic stage.^41,49-55^

Thus, the topological pathophysiology of the LGI1-associated LE allows to further elucidate the function of the subfields CA3 and the DG for hippocampal spatial memory formation in humans.^41,52,56^ Prior work from our group has shown impaired pattern separation in LGI1-LE patients, predicted by DG volume, while CA1 volume correlated with recognition memory deficits.^41^ Here, we aimed to determine the specific role of sleep on the spatial and discriminative memory performance in LGI1-LE patients in order to evaluate the impact of LGI1-associated DG/CA3 lesions on sleep-associated consolidation mechanisms.

We hypothesized that LGI1-LE patients would exhibit specific impairments in HC-dependent spatial memory compared to healthy controls, particularly in conditions that rely on pattern separation and cognitive flexibility. These deficits were expected to reflect disrupted sleep-associated consolidation mechanisms involving DG and CA3 subfields.

## Materials and methods

### Study design

**Figure 1:**
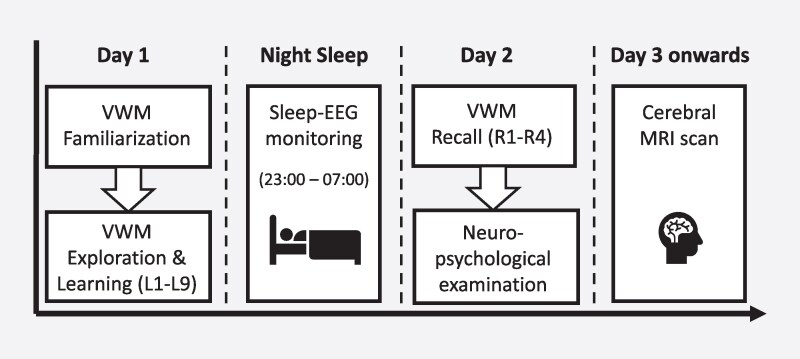
**Overall study design. Day 1 (evening presleep):** virtual Morris water maze (VWM) familiarization, exploration (L1) and learning phases (L2–L9) before bedtime at 23:00 are depicted. **Day 2 (morning post-sleep)**: waking up at 07:00, maze recall trials R1–R4, followed by neuropsychological testing. **Day 3 onwards**: cranial MRI.

A cohort of 15 patients with LGI1-associated LE (mean age: 65.13 ± 3.12 years, SEM; nine male) was examined in this study. Patients were tested on average 3.5 years (SEM 0.65) after the acute phase of their disease.^41,52^ Patients fulfilled the established diagnostic criteria for diagnosis of LGI1-LE.^51^ A 15-person healthy control group was examined and matched with no significant differences between groups in terms of sex, age and years of education. The study was conducted following the general design as depicted (Fig. 1). All sleep and cognitive assessments were conducted in participants’ home environments to maximize ecological validity and minimize disruption from unfamiliar settings. As first-night effects in sleep architecture are typically absent or substantially attenuated in home-based PSG recordings, no adaptation night was necessary in this study.^57^

The current study extends a recent study analysing visual pattern separation function in LGI1-LE patients using the mnemonic similarity task (MST).^41^ The clinical and laboratory abnormalities of these LGI1-LE patients have been described in a separate publication (Finke *et al*. 2017). The clinical outcome of the patients was in the moderate range (1.53 ± 0.26; range = 0–3) using the modified Rankin scale (mRS),^41,58^ corresponding to a mild residual functional impairment in daily life. The study was conducted according to the current state of the Helsinki Declaration of the World Medical Assembly and has been approved by the Ethics Committee of the Faculty of Medicine of the University of Kiel. Participants gave informed consent upon participation.

### Virtual Morris water maze task

#### Paradigm

The virtual Morris water maze (VWM) is a computerized spatial navigation task derived from the classic rodent-based Morris water, which assesses HC-dependent memory functions such as spatial learning, pattern separation and cognitive flexibility.^37,59-61^ Among available paradigms for human spatial navigation,^62^ we chose the VWM because it not only offers a close translational analogue to the rodent version and allows precise control of spatial cues but has also demonstrated sensitivity to hippocampal dysfunction in various clinical populations.^37,60^ Participants navigated with a first-person perspective through a virtual circular environment (island) using a joystick, after acquiring an arrangement of prominent landmarks, which served as a guide and marker of the four cardinal directions.^60,61^ Task performance was monitored across multiple phases:

Participants began with a familiarization phase training their joystick use, as well as training in the immersive nature of the task. The presleep phase comprised a single exploration trial (L1) and eight consecutive learning trials (L2–L9), testing for (i) spatial learning. The post-sleep recall phase was divided into four recall trials (R1–R4) testing (ii) recall under spatial arrangements taxing for pattern separation and pattern completion (R1–R2), (iii) place memory regarding target adherence and spatial precision (R3) and (iv) spatial reversal learning (R4). The total duration was ∼45 min for the presleep session and ∼20 min for the recall session. Participants performed the navigation task at a 24-inch LED monitor. Joystick movement speed was fixed, ensuring that differences in latency reflected cognitive rather than motor factors. The virtual environment and procedure have been previously validated in clinical populations with hippocampal dysfunction^60^ and Parkinson’s disease.^37^

#### Procedure

The task comprised consecutive phases, each including one or more trials, as detailed below:

**Familiarization phase:** Participants were introduced to the navigation mechanics by navigating through seven numbered boxes placed across the island. The phase was self-paced and ended once they demonstrated confident joystick control, typically within two runs.

**Figure 2:**
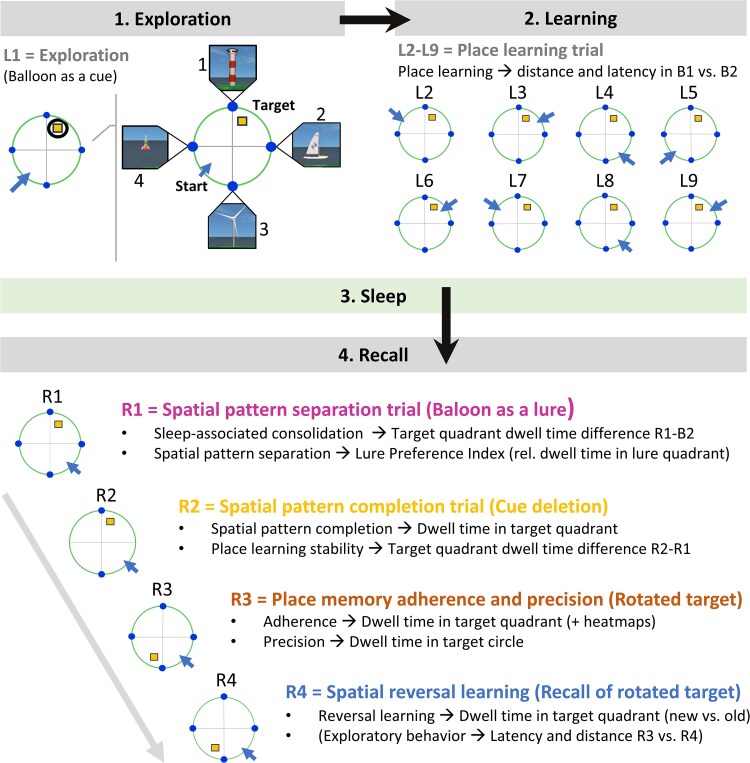
**Virtual Morris water maze (VWM) paradigm. 1-4:** depicts the principal test setting, i.e. position of the landmarks, start and target positions, and shows the specific setup of each phase. **Objects**: black circle = balloon (in L1: as cue signal; in R1: as interference signal), blue dots = distal landmarks, yellow square = target position, blue arrow = start position. The task is divided into four main phases: The familiarization phase (for visuomotor training with the joystick) is not depicted. (i) **Exploration (L1)** with a balloon as cue. (ii) **Learning (L2–L8)** of a fixed target location via variable start positions and distal landmarks (early: B1 = L2, L4; late: B2 = L7, L8). (iii) **Sleep** for memory consolidation. (iv) **Recall (R1–R4)** assessing hippocampal subfunctions: **R1**—pattern separation (balloon as lure); **R2**—pattern completion (landmark removal); **R3**—memory adherence and precision (rotated environment); **R4**—reversal learning (new target location). Coloured elements indicate task components and cognitive functions. The colours indicate the distinct functions of each trial and respective behavioural measure.

**Exploration and learning phase (L1–L9):** The learning session began with a single exploration trial (L1) in which a proximal balloon cue marked the target’s location. In trials L2–L9, this cue was removed, and participants were instructed to locate the now-hidden target using the distal landmarks. Upon reaching the correct location, they received positive auditory feedback and a reward screen. If unsuccessful after 2 min, the target was elevated for visibility to guide completion. Start positions varied between trials to encourage allocentric navigation (Fig. 2).

**Recall phase (R1–R4):** Recall was tested in the morning after sleep and comprised four trials (R1–R4) designed to selectively engage hippocampal subfunctions (Fig. 2). Each recall condition consisted of a single trial. Participants were not informed about any changes in task contingencies to minimize explicit strategy shifts. The individual trials are described below:

**Trial 1 (R1)—Place memory taxing spatial pattern separation:** A spatial lure stimulus—a balloon identical to that used in L1—was reintroduced at a novel location (left quadrant). Participants had to distinguish the original target from this misleading cue, thereby discriminating between competing spatially similar but distinct memory traces. The trial–design mirrors rodent paradigms using spatial lures to probe pattern separation^63-66^ and specifically targets the ability to resolve interference from overlapping spatial inputs,^67-70^ a process known to critically depend on the DG/CA3 subregions of the HC.

**Trial 2 (R2)—Place memory taxing spatial pattern completion:** In R2, two of the four distal landmarks were removed, while the target location remained unchanged and no proximal cue was present. This degraded-cue condition probed spatial pattern completion, requiring participants to retrieve the full spatial memory from partial input. The setup mirrors rodent paradigms using cue deletion to assess this function^71-73^ and specifically targets the CA3 subfield of the HC, known for its role in associative memory and pattern completion.^73,74^

**Trial 3 (R3)—Place memory adherence and precision:** This trial exposed the participant to a rotated target setting, similar to the classic rodent paradigm.^61^ While the distal landmarks remained stable, the target was rotated to the lower left quadrant. Participants were not informed of this change and received no specific instruction for this trial. They were simply asked to continue navigating to the previously learned target location, as in the earlier recall trials. By this setup we investigated the place memory adherence and precision (in R3).

**Trial 4 (R4)—Reversal learning:** In R4, the rotated target location from R3 was retained, and participants remained uninformed about the change. They had to independently recognize that the previously learned location was no longer valid and adjust their search strategy accordingly. This setup was designed to assess spatial reversal learning—the ability to flexibly update spatial stimulus–reward associations in response to environmental change.^33,35,63,75^ The task specifically targeted hippocampal mechanisms of cognitive flexibility, focusing on the DG–CA3 network, which is involved in detecting spatial novelty.^76,77^ The design was based on rodent maze paradigms, where shifts in target location elicit prediction errors that enhance attention to novelty and promote adaptive learning (in R4).^78-81^

#### Behavioural outcome measures

The following behavioural outcome parameters were extracted to quantify task performance across phases and respective trials:

**Place learning:** Learning performance was quantified by comparing latency (in seconds) and path length (in pool-diameter units, pd; based on an island diameter of 20 000 px^2^) between early (B1: average of L2 and L4) and late (B2: average of L7 and L8) learning phase. Thus, phases with the same starting position and sufficient run-up to the target were examined. Improved performance was reflected by reductions in both metrics from B1 to B2. Although movement speed was fixed, participants could pause or reorient without accumulating distance, making the two measures correlated but not redundant.

Path length was the primary outcome, while latency provided complementary information on navigation and pausing behaviour.

**Sleep-associated memory consolidation:** The relative dwell time in the target quadrant of the first recall condition (R1) has been set in comparison with the late learning phase B2, providing an averaged and more robust performance measure. Similar to observations in the rodent model,^82^ successful consolidation was defined as stable or increased relative to dwell time in R1.

**Place memory taxing spatial pattern separation:** To assess spatial pattern separation, we analysed relative dwell time in the lure quadrant (the adjacent left quadrant containing the balloon) during R1. Successful pattern separation was defined as the ability to reject this novel but similar spatial cue and to maintain preference for the original target quadrant.

For comparison, dwell time in the same quadrant during the final learning trial (L8) served as a neutral presleep reference to account for unspecific spatial exploration tendencies, as no lure was present during learning. Earlier trials like L7 were excluded due to positional bias in the start positions.

To quantify individual bias towards the lure location, a Lure Preference Index (LPI) was computed as *LPI = Lure* − *((100* − *Lure)/3)*, where *Lure* denotes the percentage of total dwell time spent in the lure quadrant and *(100* − *Lure)/3* represents the expected dwell time per non-lure quadrant under equal exploration (chance level = 25%). This normalization corrects for baseline exploration differences and yields a continuous measure of lure attraction relative to overall search distribution. Positive LPI values indicate a disproportionate attraction towards the lure quadrant, reflecting impaired spatial discrimination. The LPI conceptually parallels the Lure Discrimination Index (LDI) used in mnemonic similarity paradigms,^62,83^ translating response-based interference effects into a continuous spatial domain. Following prior evidence of enhanced lure interference in hippocampal dysfunction,^41,62^ we predicted higher LPI values in LGI1-LE patients relative to controls. Given this directional a priori hypothesis, a one-tailed test was used.

**Place memory taxing spatial pattern completion:** Under reduced cue conditions, relative dwell time in the target quadrant was used to assess pattern completion. Successful performance indicated accurate retrieval of the target location based on incomplete input.

We hypothesize that, in case of functional reorganization in CA3 but not DG in the post-acute LGI1 state, patients’ ability to find the target location in the presence of these degraded input signals would be restored, thus reflecting intact pattern completion.

**Place Learning Stability Index:** To quantify the persistence of spatial representations, we introduced a *Place Learning Stability Index*, building upon established findings on the persistence and reliability of spatial representations.^71,84-87^ Hippocampus subfields such as the DG and CA3 dynamically govern the adaptability of place cell firing patterns in response to spatial changes in context (i.e. remapping).^88,89^

Taking this into account, we examined participants’ performance during the rapid transition from a test environment favouring spatial pattern separation (R1) to one favouring pattern completion (R2). To quantify stability across this shift, we computed the Place Learning Stability Index as the difference in dwell time in the target quadrant (*Stability Index = dwell time in R2* − *dwell time in R1*). This index reflects how consistently spatial information was retrieved under changing conditions. Values close to zero indicated stable spatial encoding; larger deviations reflected reduced flexibility in spatial memory updating. We hypothesized that healthy participants would show stable performance across R1 and R2, while patients with LGI1-related hippocampal dysfunction would show impaired adaptation. To complement this measure, the initial heading error (IHE)—the angular deviation from the optimal path during the first 10 s—was calculated in R1 and R2 to assess allocentric navigation accuracy.^90^

**Place memory, target adherence and spatial precision:** In line with the classic rodent paradigm,^61^ place memory was assessed based on relative dwell time in the original (now incorrect) target quadrant. In that regard, target adherence was defined as dwell time in this previously learned target quadrant. Spatial precision was defined as dwell time near the original target location, operationalized as a 10% radius around its centre (*target circle*). As the target location was rotated in R3, direct comparison with R2 was not performed. Dwell time in R3 additionally served as a behavioural baseline for the subsequent reversal learning trial (R4).

**Reversal learning (R4):** Reversal learning was assessed by comparing dwell time in the new target quadrant (R4) to baseline performance in R3. Successful reversal learning was indicated by increased dwell time in the new target quadrant and, conversely, reduced dwell time in the previously learned quadrant.

#### Sleep analysis

A qualitative sleep assessment was conducted using the Pittsburgh sleep quality index (PSQI).^91^ In addition, participants underwent mobile polysomnography using the SOMNOscreen EEG 10–20 System (Somnomedics). Recordings included a three-channel electrocardiogram (ECG), electrooculogram (EOG) and electroencephalogram (EEG) based on the international 10–20 system. Signals were digitized at 128 Hz or 265 Hz and filtered at 0.2–35 Hz (EEG), 0.2–10 Hz (EOG) and 50 Hz (ECG low-pass). Analyses focused on central and frontal channels. Electroencephalogram preprocessing, power spectrum analysis and detection of conventional (fast) spindles and SOs followed a published procedure^25^ using SleepTrip (MATLAB2015a, MathWorks) and FieldTrip.^92^ Additionally, time–frequency power was computed for each detected SO in 0.5 Hz steps (0.5–30 Hz) using continuous Morlet wavelets (cycle length: frequency × 0.5, applied every 7.8 ms, time-locked to the SO turning point). Average power density (V^2^/Hz) was calculated per frequency band and channel as follows: SO (0.5–1 Hz), delta (1–4 Hz), SWA (0.5–4 Hz), theta (4–8 Hz), slow spindles (9–12 Hz) and fast spindles (12–15 Hz).

For statistical analysis, phase-locked power values were averaged across specific time windows. To examine SO–spindle coupling, a cluster-level permutation test was performed using MNE-Python’s built-in functions with correction for multiple comparisons. Electroencephalogram signals were low-pass filtered using a Savitzky–Golay filter (window size: 51; polynomial order: 3). As fast spindles predominantly occur over central regions during SO upstates, analyses focused on Cz, C3 and C4.

Sleep stages were scored offline by a trained rater according to AASM criteria.^93^ For each subject, total sleep time and time spent in stages N1, N2, SWS (N3/N4), REM sleep and movement artefacts were determined. Sleep onset was defined as the first occurrence of stage N1 followed by stage N2 after lights off. Sleep EEGs from three patients were excluded due to poor recording quality. The EEG parameters studied were most robust for the average of channels C3, C4, F3 and F4, so that only these are reported.

#### Magnetic resonance imaging

Magnetic resonance imaging was performed with 3 Tesla MRI devices (Philips Achieva). High-resolution T1-weighted images (MPRAGE; voxel size 1 × 1 × 1 mm) were acquired and processed using Freesurfer version 6 and an ex-Vivo MRI atlas. Hippocampal volumetric subfield segmentation (CA1, CA2/CA3, CA4, DG and the subiculum) was performed for the T1-weighted images according to a valid proven procedure.^41^ Structural brain volumes were normalized to estimated total intracranial volume (eTIV) to account for individual differences in head size. The clinical assessment regarding hippocampal volume loss and hippocampal sclerosis was performed by an experienced neurologist and neuroradiologist using T2-weighted and FLAIR (fluid-attenuated inversion recovery) sequences. The assessment of the MR images followed a visual consensus approach, meaning both the neurologist and neuroradiologist independently reviewed and agreed on the findings. Quantification of morphological HC changes was conducted according to the scale of the medial temporal lobe atrophy of Scheltens.^94,95^

#### Statistical analysis

The statistical analysis was carried out with the Statistical Package for the Social Sciences (SPSS) version 21. Extreme values (exceeding three interquartile distances) were excluded in a pairwise manner. Data normality was assessed using the Shapiro–Wilk test. For intergroup comparisons, normally distributed data were analysed with independent *t*-tests and nonnormally distributed data with Wilcoxon–Mann–Whitney tests. Group differences for all EEG, MRI and behavioural variables between and within the groups were checked for their significance by means of unpaired and paired *t*-test/Mann–Whitney U-test and Wilcoxon test accordingly. To examine global interaction effects, mixed-design ANOVAs were used, and in cases of nonnormality, aligned rank transformed ANOVAs (ART-ANOVAs) were applied. However, even in cases where no significant interaction was found, hypothesis-driven *post hoc* tests were still conducted.^96,97^ This approach was justified as the VWM was explicitly designed to assess hippocampal function, which is affected in LGI1-LE. The study’s *a priori* hypotheses focused on specific contrasts—such as pre- versus post-sleep changes and patient-control differences—that are critical for understanding hippocampal-dependent spatial memory dysfunction. Given the high interindividual variability in clinical populations, ANOVA may lack the sensitivity to detect subtle but theoretically meaningful effects.^96,97^ Therefore, *post hoc* analyses (*t*-tests, Mann–Whitney or Wilcoxon signed-rank test, respectively), corrected for multiple comparisons, were performed to address these targeted hypotheses and ensure alignment with the study’s objectives.^96,97^ In the case of place learning stability (R1 versus R2), we refrained from using repeated-measures ANOVA due to the high interindividual variability and instead computed a difference score (R2–R1) per subject to directly quantify the direction and magnitude of change. Dwell times were normalized to each participant’s total trial duration across all task phases to account for individual differences in exploration time. No additional normalization to the presleep baseline was applied, as direct statistical comparisons were conducted where relevant. Additionally, individual performance difference scores between relevant trials (R2 versus R1, R4 versus R3, R1 versus B2) were computed to allow for correlation analyses with EEG and MRI measures.

These differences were correlated with the MRI and EEG data averaged for both hemispheres. The relationships between two normally distributed variables were calculated with the Pearson correlation coefficient (r) and for relationships with and between two nonnormally distributed variables with the Spearman rank correlation coefficient (r_s_). For all tests, means (M) and standard errors of the mean (SEM) are reported. Statistical significance thresholds were defined as *P* < 0.05 = *, *P* < 0.01 = **, *P* < 0.001 = ***. Multiple comparisons were corrected using the Benjamini and Hochberg false discovery rate per test type and respective trial or phase.

#### Power considerations

Given the rarity of LGI1-LE, our sample size was necessarily limited (*n* = 15 per group). As an *a priori* power analysis was not feasible, we conducted a *post hoc* sensitivity analysis in G*Power 3.1 for a mixed ANOVA (group × time, two measurements, α = 0.05, ρ = 0.6, ε = 1, total *N* = 30). Under these conditions, the design provided 80% power to detect small-to-medium interaction effects (f = 0.24, ηp^2^ = 0.05), assuming a realistic within-subject correlation of ρ = 0.6 as typically observed in behavioural data.

An equivalent sensitivity analysis for a two-sample *t*-test on difference scores (α = 0.05, two-sided; power = 0.80; n_1_ = n_2_ = 15) indicated a minimal detectable effect of d = 1.06. Because within-subject correlations (ρ ≈ 0.6) reduce error variance, this estimate is conservative, and actual power for Δ-score comparisons is slightly higher. Consequently, between-group comparisons were powered primarily to detect large effects, whereas within-subject contrasts substantially increased sensitivity by minimizing interindividual variance. This power profile is typical for rare patient studies and comparable to previous work in LGI1-LE.^41,52^

## Results

### Presleep learning

**Figure 3:**
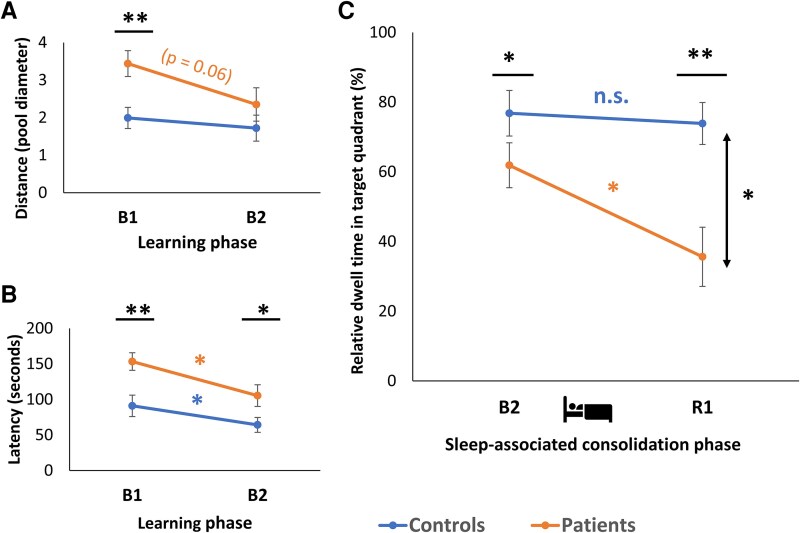
**Spatial learning and sleep-associated consolidation in the virtual water maze. A–B:** Learning performance, quantified as path length (**A**) and latency to reach the hidden target (**B**), averaged across the early learning block B1 (trials L2 and L4) and the late learning block B2 (trials L7 and L8). Both groups improved over time, as indicated by shorter path lengths and latencies in B2. However, LGI1–LE patients consistently performed worse than healthy controls, with significant group differences during learning. **C:** Sleep-associated memory consolidation, assessed as relative dwell time in the target quadrant during the late learning block B2 and the first post-sleep recall trial R1. While controls maintained stable performance across sleep, patients exhibited a significant decline in target quadrant dwell time, indicating impaired consolidation. A significant group-by-time interaction is indicated by the black asterisk on the right, reflecting a greater sleep-associated memory decline in patients compared to controls. Dwell times were normalized to each participant’s total trial duration across all task phases to account for individual differences. Orange and blue asterisks indicate significant within-group changes for LGI1–LE patients and healthy controls, respectively; black asterisks indicate significant between-group effects or group-by-time interactions. Statistical analyses included mixed-design ANOVAs with appropriate *post hoc* paired and independent *t*-tests, Wilcoxon signed-rank tests and Mann–Whitney U tests. *N* = 15 LGI1–LE patients and 15 healthy controls. ^n.s.^  *P* > 0.05; * *P* < 0.05; **P < 0.01.

As learning outcome parameters, the difference in distance (pool diameter) and latency (seconds) between the early (B1) and late learning phases (B2) was measured (Fig. 3A and B).

We observed that patients required longer paths and more time to locate the target compared to controls (Fig. 3A and B), indicating an overall deficit in spatial learning performance (*distance*: *F*(1,28) = 10.32, *P* = 0.003; *latency: F*(1,28) = 12.44, *P* = 0.002). At the same time, both groups showed a general improvement across trials, reflected in shorter path lengths and faster target acquisition from early to late learning phases (*distance: F*(1,28) = 11.91, *P* = 0.002; latency: *F*(1,28) = 10.22, *P* = 0.003). Despite differences in overall performance and improvement over time, the interaction between both groups and phases was not significant, suggesting similar learning trajectories in both groups (*distance: F*(1,28) = 3.02, *P* = 0.093; *latency: F*(1,28) = 2.58, *P* = 0.120). Despite the absence of a significant global interaction, targeted *post hoc* analyses were conducted to address the study’s objectives, as outlined in the Methods section.

In B1, patients performed significantly poorer compared to controls, with longer distances and latencies to the target (*distance: t*(28) = −3.248, *P* = 0.003; *latency: t*(28) = −3.196, *P* = 0.003). In B2, the group difference in latency persisted, while the difference in distance was no longer statistically significant (*distance: U* = 74.5, *P* = 0.120; *latency: U* = 57, *P* = 0.041). When examining learning progress across trials, both controls and patients reduced their latencies significantly (*controls: W* = 96, *z* = 2.045, *P* = 0.041; *patients: W* = 99, *z* = 2.215, *P* = 0.041). For path length, only patients showed a numerical reduction, which did not reach statistical significance after correction for multiple testing (*controls: W* = 72, *z* = 0.682, *P* = 0.524; *patients: W* = 98, *z* = 2.158, *P* = 0.060). Although the prior nonsignificant interaction effect indicated comparable learning trajectories, further analyses revealed that patients initially performed worse than controls and showed a trend towards greater improvement in path length. This pattern suggests that LGI1 patients were able to successfully encode spatial information and benefit from repeated learning trials (Fig. 3A and B).

### Post-sleep recall

#### Trial 1—place memory taxing pattern separation

##### Measure of sleep-associated consolidation of place memory (R1–B2)

To assess sleep-associated memory consolidation, we examined changes in dwell time in the target quadrant from the late learning phase (B2) to the first post-sleep recall (R1), as shown in Fig. 3C. Successful consolidation was defined as stable or increased dwell time in the target quadrant after sleep, reflecting reinforced allocentric place memory.

Patients showed a greater decline in spatial memory performance across sleep compared to controls, as reflected in a significant differential change over time between groups in dwell time in the target quadrant (*F*(1,28) = 5.21, *P* = 0.031). While patients dwelled significantly less in the target quadrant, compared with their prior performance in B2 (*W* = 18, *z* = −2.385, *P* = 0.015), the control group maintained a stable performance (*W* = 39, *z* = −1.193, *P* = 0.252). This effect remained significant when comparing the average of both early recall trials (R1 + R2) to presleep performance in patients, confirming a decline in spatial memory across sleep despite the varying task demands (*M* = 41.93 ± 6.95; *t*(14) = 2.522, *P* = 0.024). A direct between-group comparison in R1 further confirmed reduced dwell time in patients relative to controls (*U* = 188, *P* = 0.004).

To address the possibility that poorer encoding might explain the reduced recall in patients, we performed an ANCOVA predicting post-sleep recall (R1) from group while controlling for presleep learning performance (B2 dwell time). The main effect of group remained significant (*F*(1,27) = 9.66, *P* = 0.004), indicating that the reduced recall in LGI1-LE patients cannot be explained solely by weaker encoding. Taken together, these findings indicate impaired sleep-associated consolidation of hippocampal place memory in LGI1 patients (Fig. 3C).

##### Place memory performance in spatial arrangements taxing pattern separation (R1)

During the first post-sleep recall (R1), a salient spatial lure (balloon) was introduced in the quadrant adjacent to the previously learned target location to probe pattern separation. Successful performance required rejecting this similar but novel cue and maintaining preference for the original target quadrant.

Patients showed a stronger bias towards the lure quadrant than controls (main effect of group: *F*(1,28) = 8.34, *P* = 0.007), indicating increased susceptibility to spatial interference. Before sleep, when no lure was present (L8), both groups explored the same quadrant at comparable levels (*U* = 72, *P* = 0.153), confirming that this bias emerged specifically in response to the lure manipulation rather than preexisting spatial preferences.

**Figure 4:**
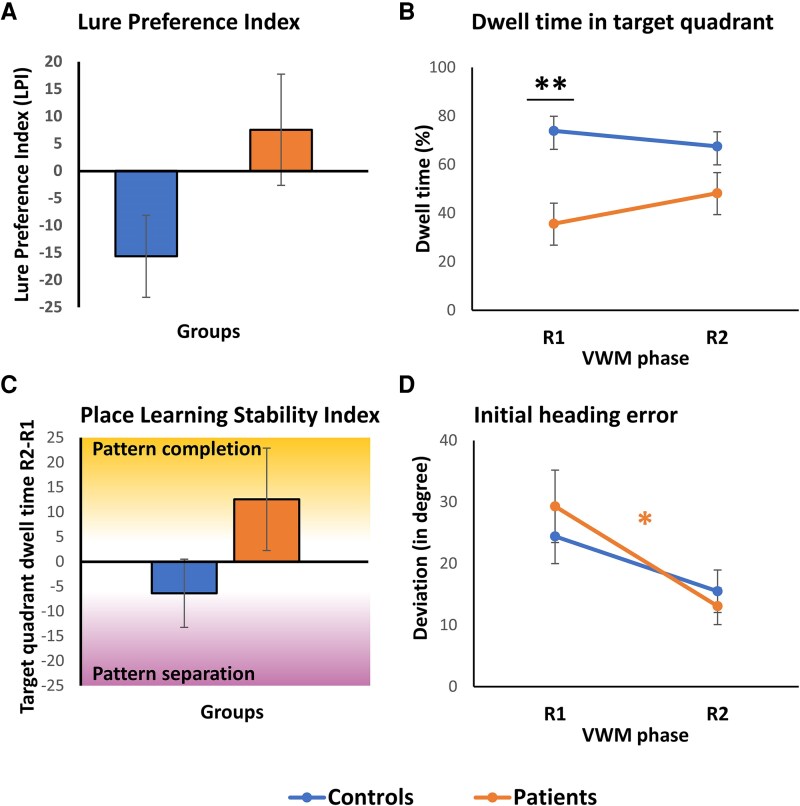
**Behavioural measures of pattern separation, pattern completion and place memory stability. A:** Lure Preference Index (LPI) in R1. Positive values indicate a stronger bias towards the lure quadrant relative to chance. Patients showed significantly higher LPI values than controls (*W* = 66.5, *P* = 0.026), reflecting increased lure attraction and reduced hippocampal pattern separation. Statistical comparison between groups was performed using a Mann–Whitney U test (one-tailed). **B:** Place Learning Stability Index (PLSI), calculated as the difference in relative target quadrant dwell time between R2 and R1 (R2–R1). Positive values indicate a relative shift towards pattern completion, whereas negative values indicate a stronger pattern separation bias. **C:** Relative dwell time in the target quadrant during R1 and R2. **D:** Initial heading error (IHE) during R1 and R2. Data are shown as group means ± SEM. Black asterisks indicate significant between-group differences; orange asterisks indicate significant within-group changes in LGI1–LE patients. Statistical analyses included independent and paired *t*-tests as well as Mann–Whitney U tests. *N* = 15 LGI1–LE patients and 15 healthy controls. *P < 0.05; **P < 0.01.

To capture lure attraction independent of baseline exploration, a LPI was computed (LPI = lure − ((100 − lure)/3)). As expected, patients showed significantly higher LPI values than controls (*W* = 66.5, *P* = 0.026, one-tailed according to directional hypothesis based on prior evidence; see Methods section), corresponding to a medium effect (r_rb_ = 0.41, SE = 0.21) in the expected direction. This pattern indicates impaired hippocampal pattern separation in LGI1-LE, reflected in a disproportionate attraction towards similar spatial cues (Fig. 4A).

#### Trial R2—place memory performance taxing pattern completion

##### Place memory performance in a pattern completion—favouring spatial arrangement

In this post-sleep recall trial R2, the application of place memory in a pattern completion favouring environment was tested by means of a cue deletion task. While in R1 the patients dwelled significantly less than the controls in the target quadrant, in R2 both groups did not dwell differently (*patients:* 48.212 ± 8.816; *controls:* 67.491 ± 7.609; *U* = 115; *P* = 0.115). This documents a similar performance of place memory taxing spatial pattern completion function between both groups (Fig. 4B).

##### Place Learning Stability Index (dwell time difference R2–R1)

We calculated the difference in dwell time between R2 and R1 as an individual stability index. This index reflects how consistently spatial memory was applied across changing spatial contexts. Patients showed numerically higher stability scores than controls (*patients*: 12.57 ± 10.32; *controls*: −6.38 ± 6.87; *t*(28) = −1.528, *P* = 0.138; Fig. 4C). Although this difference did not reach statistical significance, the observed effect size (Cohen’s d = 0.56) suggested a moderate group difference. A *post hoc* power analysis (G*Power 3.1; two-tailed independent-samples *t*-test, α = 0.05, n_1_ = n_2_ = 15, d = 0.56) yielded an achieved power of 0.32, indicating that the study was underpowered to detect this effect. This result should therefore be regarded with caution.

To support this finding, we analysed the IHE as an additional behavioural marker (see Methods section). Patients significantly reduced their IHE from R1 to R2 (*R1:* 29.293 ± 5.881; *R2*: 13.073 ± 2.994; *t*(14) = 2.453, *P* = 0.028; Fig. 4D), indicating more accurate navigation when fewer spatial cues were available. The direction and specificity of this change are consistent with a shift towards pattern completion mechanisms. Thus, spatial navigation might have become more dependent on the discriminative quality of the input signals, whereas when faced with a degraded set of spatial cues (R2), patients succeeded.

#### Trial R3—measure of place memory adherence and precision

**Figure 5:**
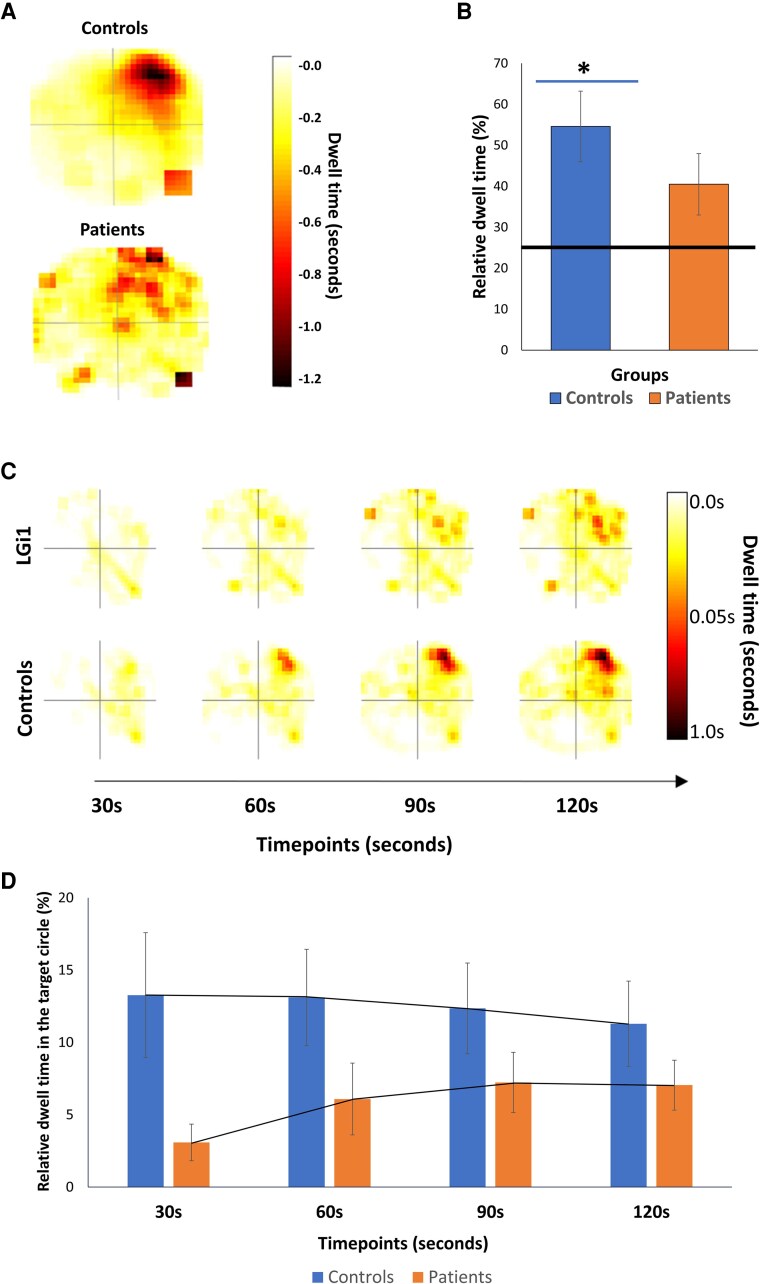
**Measure of place memory adherence and precision (R3). A: Dwell time** in controls (upper panel) and LGI1 patients (lower panel) are shown as colour-coded heatmaps at the timepoint of 120 s (Gaussian smoothing kernel Type 5 was used). The colour scale indicates accumulated dwell time within each spatial bin. A more inhomogeneous distribution of the relative dwell times for the patients can be seen. **B:** Group comparison of the relative dwell time in the learned target quadrant during R3 reflecting target adherence. Bars represent mean ± SEM. The dashed line indicates performance at the level of chance (25%). The asterisk marks a significant difference from chance level for the control group (*P < 0.05; one-sample test). **C:** Dwell time at consecutive time points for controls (upper panel) and LGI1 patients (lower panel). Controls entered and remained in the target area earlier and more consistently than patients. Start location: lower right quadrant; learned target: upper right quadrant. **D:** Spatial precision shown is the dwell time (mean ± SEM) within the target circle (10% of island area) as a marker of spatial precision at consecutive time points. Controls showed higher precision by spending more time near the target, whereas patients reached the area later and less reliably. Statistical analyses included Mann–Whitney U tests. *N* = 15 LGI1–LE patients and *N* = 15 healthy controls.

In R3, place memory was assessed in a rotated spatial setting, equivalent to the classic rodent paradigm.^61^ Controls demonstrated intact place memory regarding target adherence, spending significantly more time in the original target quadrant than expected by chance (i.e. *>25% of trial time*, *M* = 54.59 ± 8.62; *W* = 103; *P* = 0.024). In contrast, patients showed a weaker preference for the previously learned location (*M* = 40.48 ± 7.50; *t*(14) = 2.063; *P* = 0.058), suggesting reduced retention of the spatial representation (Fig. 5A and B).

Regarding spatial precision, heatmaps revealed that controls approached the target more directly, whereas patients reached the area later and via less efficient paths. This impression was supported by dwell time in the defined target circle (10% of the island area), where only controls showed a marked tendency to remain close to the original location (Fig. 5C and D). Taken together, these findings suggest reduced adherence and lower spatial precision in LGI1 patients, pointing to a less stable and less accurate representation of the learned spatial target compared to healthy controls.

#### Trial R4—spatial reversal learning

**Figure 6:**
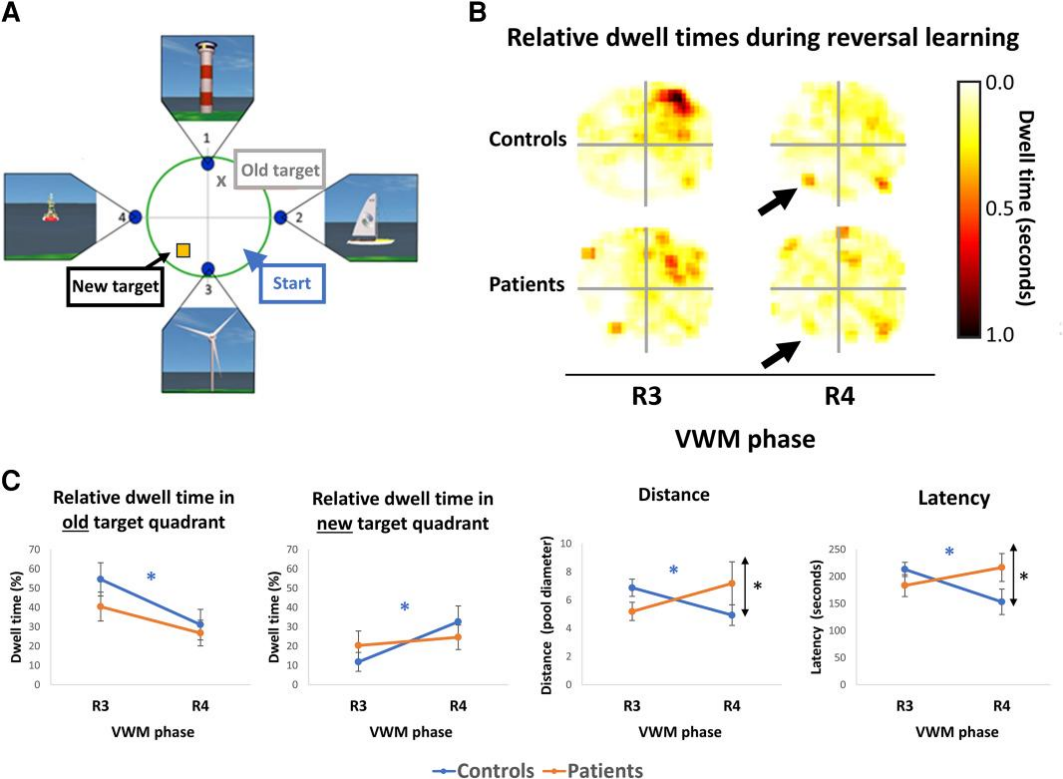
**Reversal learning. A: Design of the reversal trial (R4).** The target remained in the rotated location from R3. Participants were not informed of this change and had to adapt to the new target position. Depicted are the old and new target positions, as well as the start and landmark positions. **B:** Time-resolved dwell times in the rotated target quadrant during R3 and R4 for controls (upper panel) and LGI1 patients (lower panel) are colour-coded (right, in seconds) depicted. The time slot was selected for 90 s. The arrow marks the location of the new target quadrant in R3 and R4. **C:** Behavioural measures of reversal learning. Shown for both groups across R3 and R4 are dwell times in the old and new target quadrant, as well as latency (time until reaching the target or trial ended) and distance (total path length), reflecting general search behaviour. Note the crossing lines in dwell time trajectories and the opposite patterns of change in latency and distance between groups. Statistical analyses included mixed-design ANOVA and *post hoc* paired *t*-tests and Wilcoxon signed-rank tests. Data are shown as mean ± SEM. *N* = 15 LGI1–LE patients and 15 healthy controls.; * *P* < 0.05.

To assess reversal learning, the target remained in the rotated quadrant in R4, prompting participants to adjust to the new spatial configuration (Fig. 6). Across both groups, respectively, dwell time in the new target quadrant increased (*F*(1,28) = 5.02, *P* = 0.033), and time in the old quadrant decreased (*F*(1,28) = 9.35, *P* = 0.005) from R3 to R4. While no group effect emerged, *post hoc* analyses revealed that only controls adapted their navigation accordingly: they spent more time in the new target quadrant (*W* = 9; *z* = −2.134; *P* = 0.037) and less in the old one (*W* = 91; *z* = 2.417; *P* = 0.034). Patients, in contrast, showed no change in their quadrant preference across trials (*old target*: *t*(14) = 2.098; *P* = 0.110; *new target*: *W* = 27; *z* = −0.941; *P* = 0.367). This pattern was supported by additional measures: latency and path length changed over time differently across groups (*latency*: *F*(1,27) = 5.56, *P* = 0.026; *distance: F*(1,28) = 6.74, *P* = 0.015), but only controls improved their navigation, reaching the new target faster (*t*(14) = 2.47, *P* = 0.027) and via shorter distance (*t*(14) = 2.50, *P* = 0.027). Patients showed no such improvement (*latency: t*(13) = −1.63, *P* = 0.256; *distance: W* = 48, *z* = −0.68, *P* = 0.524), suggesting a less goal-directed behaviour, indicating unsuccessful reversal learning (Fig. 6).

### Sleep architecture and electroencephalogram oscillatory measures

The cross-modal design of our study enabled correlational analyses linking behavioural, EEG and MRI measures. As such, these analyses are inherently exploratory in nature and are interpreted with caution given the limited sample size due to rarity of the LGi1-LE.

#### Sleep quality and macro-architecture of sleep

There was no significant difference between the patients and their healthy control subjects regarding the PSQI (*patients:* 5.15 ± 0.73; *controls:* 3.00 ± 0.62; *U* = 157; *P* = 0.670). However, the mean PSQI of the LGi1 patients was at the threshold for clinically relevant sleep disturbances, which we considered in the interpretation of the behavioural data.

Importantly, subjective sleep quality as assessed by the PSQI did not correlate with spatial task performance (all *P*-values > 0.349; after correction for multiple testing using the Benjamini–Hochberg procedure, all *P* = 0.739). Regarding the sleep times (time in bed, sleep onset, sleep duration) as well as the EEG analysis of the macro-architecture of sleep (sleep stages), the patients’ night sleep was not significantly different as compared to the healthy control group (Table 1).

**Table 1:** Results of the sleep analysis

Criteria of analysis	LGI1 patients	Control group	F	*P*
**Sleep time**				
Total sleep time (min)	450.00 ± 17.40	433.67 ± 17.49	0.48	0.497
Latency of sleep onset (min)	101.46 ± 15.86	68.67 ± 13.75	2.50	0.191
Time in bed (h)	7.94 ± 0.23	7.39 ± 0.25	2.59	0.191
**Sleep stages**				
N1 (%)	9.24 ± 0.91	8.03 ± 0.94	0.85	0.734
N2 (%)	39.99 ± 3.73	41.31 ± 3.04	0.08	0.925
SWS (%)	15.00 ± 0.94	16.50 ± 2.26	0.23	0.925
REM (%)	13.35 ± 1.98	19.19 ± 1.98	5.42	0.132
Wake (%)	18.50 ± 3.08	11.22 ± 1.93	4.51	0.132
Movement (%)	3.87 ± 2.16	3.76 ± 1.55	0.01	0.925
**Density (per30 s)**				
Spindle (12–15 Hz)	1.74 ± 0.26	1.41 ± 0.18	1.09	0.308
SO (0.5–1 Hz)	0.87 ± 0.15	0.86 ± 0.10	<0.01	0.971
**Power density (µV^2^/Hz)**				
SO (0.5–1 Hz)	114.40 ± 19.48	124.64 ± 18.29	0.14	0.714
Delta (1–4 Hz)	23.45 ± 2.87	26.94 ± 4.02	0.45	0.510
SWA (0.5–4 Hz)	35.15 ± 4.90	38.15 ± 5.88	0.14	0.713
Theta (4–8 Hz)	4.19 ± 0.87	5.75 ± 1.23	0.96	0.337
Slow spindles (9–12 Hz)	1.77 ± 0.29	1.83 ± 0.28	0.04	0.851
Fast spindles (12–15 Hz)	1.44 ± 0.26	1.14 ± 0.16	1.09	0.307

N1 = sleep stage 1, N2 = sleep stage 2, SWS = slow-wave sleep, REM = rapid eye movement sleep. The following are given: MW ± SEM; F (1; 24). Densities and power densities are averaged over central (C3, C4) and frontal (F3, F4) channels.

#### Sleep-associated EEG oscillations and microarchitecture of sleep

We examined spindle and SO density and average power densities (V^2^/Hz) for the frequency bands of SO, Delta, SWA, theta, slow spindles and fast spindles. Regarding the latter frequency bands, no differences in average power densities were found between the cohorts (Table 1). Furthermore, there were no differences regarding the SO density or regarding the spindle density (Table 1).

**Figure 7:**
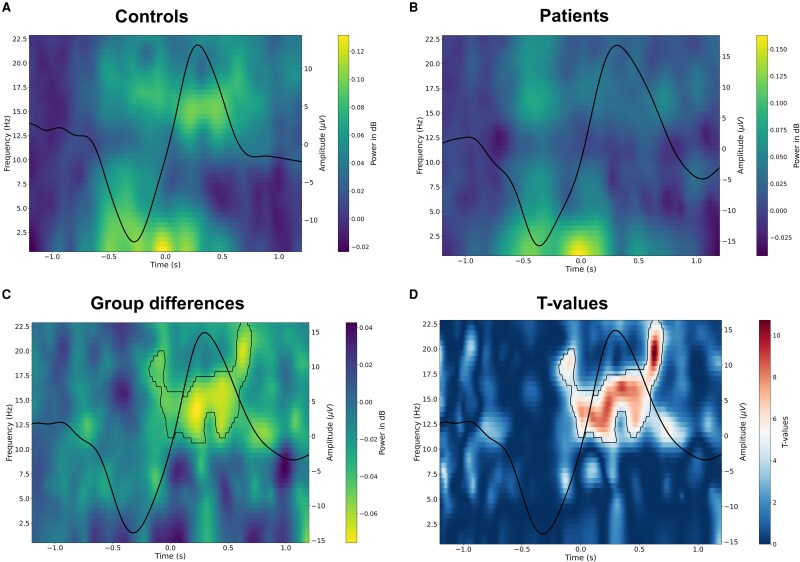
**Time–frequency coupling analysis. A** depicts the averaged time–frequency plot of EEG wavelet power during slow oscillations for control subjects (*n* = 14). **B** shows the equivalent plot for LGI1–LE patients (*n* = 12). Both are displayed within a ± 1.2 s time window centred on the slow oscillation turning point (0 s) and across a frequency range of 0–30 Hz. **C** displays the difference between patients and controls in time–frequency coupling over central channels. Note: the enhanced spindle power during the SO upstate (0 to +500 ms) in the 10–16 Hz frequency range. Panels A–C show descriptive representations without statistical testing. **D** illustrates the T-values of the time–frequency comparison between groups. Statistical significance was assessed using cluster-based permutation testing based on t-statistics. *N* = 15 LGI1–LE patients and 15 healthy controls. Despite the notable patterns, statistical significance was not reached (*P* = 0.1).

A consecutive time–frequency analysis (Fig. 7) revealed increased theta (4–8 Hz) and delta (0.5–4 Hz) power following the SO turning point in both groups. While theta is thought to modulate spindle bursts, delta includes the SO itself. Interestingly, only the healthy control group, but not the patients, showed a marked increase in fast spindle power (10–16 Hz) during the SO upstate (0 to +500 ms post-turning point). This timing highlights the phase-specific coupling between fast spindles and the SO’s upstate. In LGI1-LE patients, fast spindle activity appeared more diffusely distributed and less temporally aligned with the excitatory phase of the SO. A cluster-based permutation analysis (Fig. 7D) showed elevated T-values in the same 10–16 Hz band during the excitatory phase. Statistical significance was not reached after cluster-level correction for multiple testing (*P* = 0.1). Therefore, these descriptive findings should be interpreted cautiously but may be consistent with the hypothesis that LGI1-LE-related hippocampal dysfunction disrupts the precise timing of hippocampal output (e.g. SWRs) during SO cycles.

#### Correlations between EEG oscillations and behavioural measures of the VWM

##### Correlations with place memory performance

**Figure 8:**
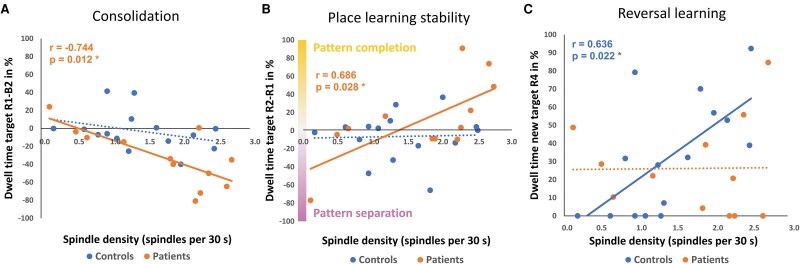
**Correlations between spindle density and spatial navigation. A:** depicts the patients’ correlation between spindle density and the consolidation measure (target dwell time R1–B2 in %); note that those patients with higher spindle density performed relatively worse the post-sleep-condition R1. **B:** shows the correlation between the measure of Place Learning Stability Index (target quadrant dwell time R2–R1) and the spindle density in the patients. The yellow/purple column marks the spectrum from pattern separation in R1 to pattern completion in R2. **C:** depicts the positive correlation between the measure of reversal learning (dwell time in the new target quadrant in R4) with the spindle density in the controls. Each data point represents one individual participant (orange = LGI1–LE patient, blue = healthy control). Pearson correlation analyses were performed. *N* = 12 LGI1–LE patients and 15 healthy controls. Bold regression lines indicate statistically significant correlations within the respective group (*P < 0.05); dotted lines are for visualization only and are not significant.

In patients, higher spindle density correlated negatively with the change in dwell time in the target quadrant from pre- to post-sleep (*B2–R1: r* = −0.744, *P* = 0.012; Fig. 8A) while showing no association with R1 or B2 individually. Furthermore, spindle density was positively associated with the Place Learning Stability Index, reflected by increased dwell time from R1 to R2 (*r* = 0.686; *P* = 0.028; Fig. 8B). In line with this, patients also showed reduced latencies towards the target between R1 and R2 (*r* = −0.677; *P* = 0.016).

Considering the previously reported negative correlation between spindle density and changes in dwell time, and given the inherently exploratory nature of correlational analyses, these findings suggest that higher spindle density alone may not necessarily translate into better post-sleep retrieval performance in LGI1 patients.

##### Correlations with post-sleep reversal learning performance

In control subjects, higher spindle density was associated with improved reversal learning, as indicated by increased dwell time in the new target quadrant during R4 (*r* = 0.636; *P* = 0.022; Fig. 8C). Control subjects with higher spindle density adapted more efficiently to the change in target location: they reached the new target position faster (r_s_ = −0.630; *P* = 0.028) and via shorter distance (*r* = −0.616; *P* = 0.028). A similar association was observed for the SO density, too (distance R4: *r* = −0.521; *P* = 0.046). For the LGI1–LE patients, we did not see any relationship regarding reversal learning. The power density of relevant frequency bands was not significantly correlated with place learning or with reversal learning. However, these findings should be interpreted in light of the limited sample size, reflecting the rarity of the disease.

### MRI analysis

#### Hippocampal volumetry

**Figure 9:**
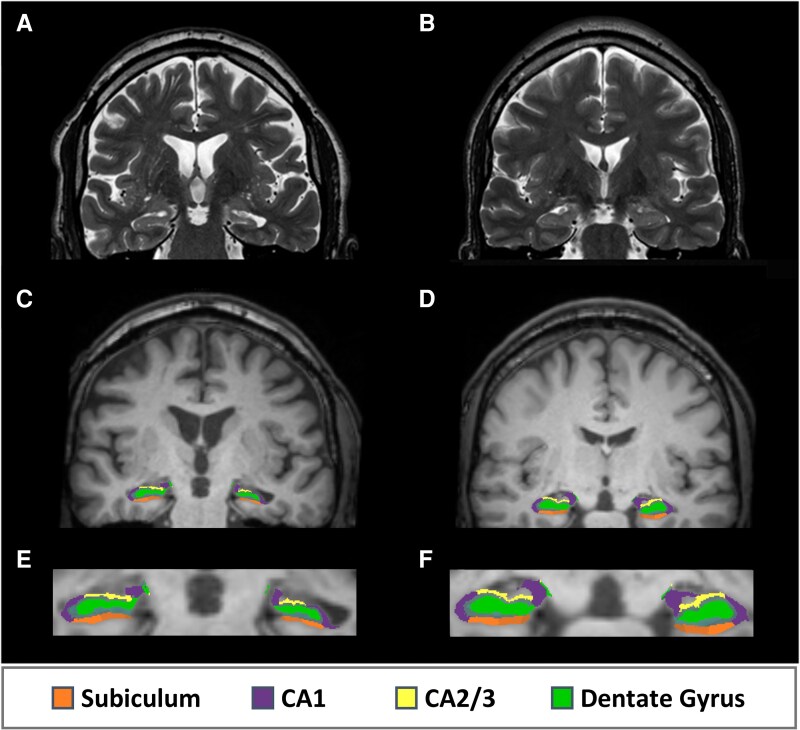
**MRI image of hippocampal atrophy of the patients.** Cranial MRI images of one representative LGI1–LE patient (left row: **A**, **C**, **E**) and a matched control participant (right row: **B**, **D**, **F**). **A** and **B**: T2; **C** and **D**: T1 with marked HC regions for volumetric analysis. **E** and **F**: T1 magnification. The volumetry of a LGI1 patient shows bilateral hippocampal atrophy. Note: the hippocampal sclerosis is accompanied by an internal structural loss primarily of the layers for CA3 and DG.

The total hippocampal volume and the volumes of its subregions (subiculum, CA1, CA3, DG), all averaged across hemispheres, were significantly reduced in the patient group (Table 2). The differences described were more pronounced on the right side than on the left. In this sense, global hippocampal atrophy was diagnosed in 13 and DG-related Ammon’s horn sclerosis in nine out of 14 of these clinical patients’ MRIs^41^ (Fig. 9). The group difference in hippocampal volume was paralleled by a significant reduction in total grey matter volume, whereas eTIV did not differ between groups (Table 2).

**Table 2:** Group differences regarding hippocampal subfield volumes

Region	Patients	Controls	*F*	*P*
eTIV	1 461 938 ± 64 934	1 446 395 ± 51 140	0.017	0.898
Total grey matter	566 232 ± 14 202	610 232 ± 11 129	6.02	0.021*
HC	2883 ± 112	3381 ± 96	11.68	0.002**
CA1	545 ± 21	641 ± 18	11.78	0.002**
CA3	182 ± 8	219 ± 6	12.32	0.002**
DG	467 ± 20	554 ± 16	11.72	0.002**
Subiculum	354 ± 14	425 ± 14	13.49	0.002**

The volumes shown are given as bilateral MW ± SEM (mm^3^) and have been normalized to the estimated total intracranial volume (eTIV); F (1; 27); *P* values are corrected for six comparisons (eTIV excluded, due to sequential testing), * *P* < 0.05; ** *P* < 0.01; *** *P* < 0.001.

#### Correlations with hippocampal volumetry

##### Correlations between HC volumes and consolidation of place memory (R1–B2)

**Figure 10:**
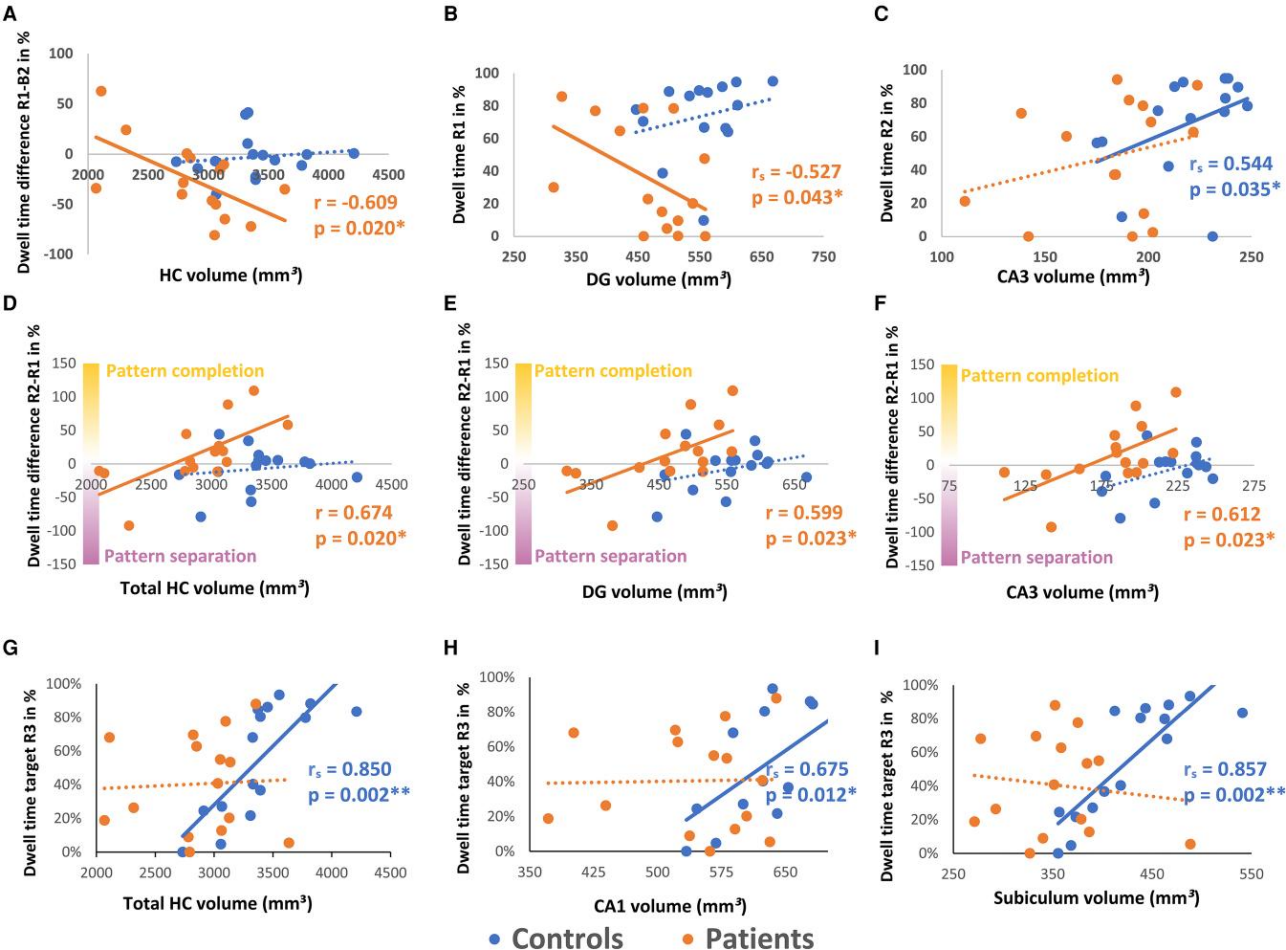
**Correlations between hippocampal volumes and spatial memory consolidation, retrieval, stability and target adherence. A:** Correlation between total hippocampal volume (HC) and sleep-associated spatial memory consolidation, expressed as the difference in relative target quadrant dwell time between the final learning phase (B2) and the first recall trial (R1; R1–B2). **B:** Correlation between dentate gyrus (DG) volume and relative dwell time in the target quadrant during the pattern separation (PS)-favoured trial (R1). **C:** Correlation between CA3 volume and relative dwell time in the target quadrant during the pattern completion (PC)-favoured trial (R2). **D–F:** Correlations between total HC (**D**), DG (**E**) and CA3 (**F**) volumes and the Place Learning Stability Index, calculated as the difference in relative target quadrant dwell time between R2 and R1 (R2–R1). The yellow/purple column marks the spectrum from pattern separation in R1 to pattern completion in R2 as a further visual reference for the stability marker R2–R1. Please note that patients’ performance is relatively shifted towards pattern completion, resulting in a functional disequilibrium. **G–I:** Correlations between total HC (**G**), CA1 (**H**) and subiculum (**I**) volumes and target adherence during the rotated target trial (R3), quantified as relative dwell time in the originally learned target quadrant. Each data point represents one participant (orange = LGI1–LE patient; blue = healthy control). Solid regression lines indicate statistically significant correlations; dotted lines are shown for visualization only. Pearson correlations were performed for panels **A** and **D–F**. Spearman correlations were performed for panels **B**, **C** and **G–I**. *N* = 15 LGI1–LE patients and 15 healthy controls. The principal hippocampal volume–behaviour relationships are shown. Additional hippocampal subfield analyses corresponding to the consolidation and place memory stability are provided in the Supplementary material. Correlation analyses for consolidation (**A**), place memory stability (**D–F**) and target adherence (**G–I**) were corrected for five hippocampal volume comparisons. Panels **B** and **C** depict the prespecified (*a priori* hypothesis-driven) analyses of DG-dependent pattern separation (R1) and CA3-dependent pattern completion (R2), respectively. As only the predefined target subfield (R1 = DG; R2 = CA3) was tested in each analysis, no correction for multiple comparisons was applied (see Methods). *P < 0.05; **P < 0.01.

Interestingly, for the patient cohort, the hippocampal volumes correlated negatively with the difference in relative target quadrant dwell time between the pre- and post-sleep phases (Fig. 10A). Thus, in patients, a relatively larger HC correlated with a greater performance decrement over sleep, thus, surprisingly, suggesting a lesser stable memory consolidation (Fig. 10A; subfield-specific correlations are shown in Supplementary Fig. S1).

This finding was further supported when examining R1, respectively, demonstrating that particularly those patients with a relatively larger HC and especially larger DG volume performed worse (Fig. 10B). Additionally, this correlation was also seen regarding a prolonged latency and longer pathway to the target in R1.

For the controls, on the other hand, a larger HC volume correlated positively with a longer dwell time in the target quadrant during both learning (B2) and recall (R1, R2) (Fig. 10C for CA3 volume correlation), but not for the sleep-associated consolidation measure (diff R1–B2) and neither for the difference between R2 and R1. Additionally, a larger subiculum was correlated with a smaller value for the pathway difference B2–B1, indicating a shortening of the travelled distance over the learning blocks in those controls with a relatively larger HC volume.

##### Correlations between HC volumes and place memory stability (R2–R1)

Regarding the patients, a larger volume of total HC and CA1, CA3, and DG was associated with an increasing relative dwell time from R1 to R2 (difference R2–R1) (Fig. 10D–F; additional CA1 subfield correlation is provided in Supplementary Fig. S2). Those patients showed themselves to be stable in their place learning under the cue deletion (R2) and were able to improve, starting from their recall baseline (R1).

##### Correlations between HC volumes and place memory, adherence and spatial precision (R3)

In the control group, volumes of the CA1 region, subiculum, and total HC correlated positively with place memory performance in terms of target adherence, as indicated by longer dwell times in the learned target quadrant in R3 (Fig. 10G–I). Spatial precision, as indicated by relative target circle dwell time (at consecutive time windows, i.e. 60 and 120 s) was positively correlated with same volumes (*HC: 60 s*: *r* = 0.812; *P* = 0.002; *120 s*: *r* = 0.718; *P* = 0.008; *subiculum: 60 s*: *r* = 0.774; *P* = 0.002; *120 s*: *r* = 0.751; *P* = 0.005; *CA1: 60 s*: *r* = 0.771; *P* = 0.002; *120 s*: *r* = 0.687; *P* = 0.008). These findings suggest that greater volumes in these regions support more precise spatial memory retrieval in healthy individuals. No such correlations were observed in the patient group, underscoring the specific structure–function relationship for successful place learning in controls.

##### Correlations between HC volumes and reversal learning

For the controls, we observed an inverse correlation between the dwell time difference R4–R3 for the old target quadrant and the volume of the subiculum (*r* = −0.646, *P* = 0.028) and for the HC itself (*r* = −0.693, *P* = 0.025). This indicates that especially those control subjects with a relatively large HC and subiculum showed a more pronounced reversal learning.

For the patients, there were no significant correlations with reversal learning.

### Neuropsychological examination

Patients exhibited impairments in attention, visuoconstructive memory, short-term and working memory, executive function, verbal memory and verbal fluency (Table 3). To test whether reduced executive or working-memory abilities contributed to the navigation deficits, neuropsychological measures (TMT, digit span total, ROCF copy and recall) were correlated with key behavioural outcomes from the spatial task. In patients, none of the executive measures correlated significantly with navigation performance (all *P* > 0.13; adjusted *P* > 0.52). The same held for controls, except that visuoconstructive performance (ROCF copy) correlated positively with target dwell time (*r* = 0.65, *P* = 0.008) and negatively with lure dwell time (*r* = −0.61, *P* = 0.017); both survived correction (adjusted *P* = 0.032 and 0.034). Importantly, controlling for TMT-B or general executive ability did not alter the significant group × time effects, indicating that the spatial memory impairments in LGI1–LE are unlikely explained by executive dysfunction.

**Table 3:** Neuropsychological data of both groups

Test		LGI1 patients	Control group	T/U	*P*
RAVLT^a^	Learning (Sum 1-5)	36.20 ± 4.46	60.67 ± 1.97	5.022	<0.001***
	Immediate recall (6)	6.33 ± 1.41	13.60 ± 0.46	30^e^	<0.001***
	Delayed recall (7)	5.87 ± 1.46	14.07 ± 0.36	25^e^	<0.001***
	Recognition	10.00 ± 1.13	14.60 ± 0.16	33^e^	<0.001***
TMT^b^	A	57.13 ± 8.28	35.87 ± 4.22	169^e^	0.038*
	B	201.13 ± 47.94	95.23 ± 14.69	160.5^e^	0.045*
ROCF^b^	Copy	31.80 ± 1.20	29.40 ± 1.42	141.5^e^	0.233
	Recall	15.77 ± 2.50	28.67 ± 1.38	4.514	<0.001***
Digit Span^c^	anterograde	6.80 ± 0.63	8.53 ± 0.52	2.14	0.041*
	Retrograde	5.00 ± 0.67	7.93 ± 0.63	3.20	0.009**
	Total	11.80 ± 1.21	16.47 ± 1.03	2.93	0.001**
RWT^d^	‘Surnames’	20.47 ± 1.98	30.60 ± 1.91	38.00^e^	0.002**
	‘S’	12.40 ± 1.61	18.20 ± 1.32	2.78	0.010*
	‘K’	11.33 ± 1.76	20.00 ± 1.70	30.5^e^	0.001**
	‘Animals’	16.93 ± 2.36	27.47 ± 2.54	3.04	0.007**

ROCF, Rey–Osterrieth Complex Figure Test; TMT, trail-making test A and B (average values given in seconds); RWT, Regensburg Word Fluid Test. *N* = 15 LGI1–LE patients and 15 healthy controls. Indicated are M ± SEM of the achieved scores; *t*(28) values are reported; U values (Mann–Whitney U test) are indicated by ^e^; *P*-values were corrected for multiple comparisons: ^a^seven comparisons; ^b^two comparisons; ^c^three comparisons; ^d^four comparisons. The multiple-choice vocabulary intelligence test of one patient (non-native German speaker) was missing. * *P* < 0.05; ** *P* < 0.01, *** *P* < 0.001.

## Discussion

The LGI1-associated LE is a hippocampal lesion model as these patients suffer hippocampal damage of the DG and CA3 region. To assess the effects of this DG and CA3 network disruption, we analysed spatial memory performance, hippocampal subfield volumes and sleep parameters in patients and healthy controls. Our aim was to investigate how this disruption impacts spatial learning, sleep-associated consolidation and retrieval and cognitive flexibility during reversal learning. We found that LGI1–LE patients exhibit significantly impaired post-learning consolidation of representations, particularly apparent in recall conditions requiring spatial pattern separation. The patients, moreover, demonstrated less precise spatial memory and failed in reversal learning. In addition to these robust measures, exploratory analyses—including descriptive SO–spindle coupling patterns and cross-modal correlations—were conducted in a hypotheses-generating manner.

### Sleep in LGI1–LE

Although mean PSQI values of LGI1–LE patients were slightly higher than those of controls, they remained at the borderline of the clinical threshold^91^ and did not differ significantly between groups. Moreover, our LGI1-LE patients showed similar sleep architecture and sleep-associated oscillations as healthy controls. This contrasts with previous reports of disturbed sleep and altered spindle activity in the acute phase of the disease.^55,98-101^ Improvements following immunotherapy support the view that antibody-mediated alterations in neuronal excitability in regulatory sleep-related networks might be the main cause for such sleep impairments.^55,98^

In our study, sleep was assessed at 2.67 ± 0.53 years after the initial diagnosis, providing a long-term perspective on residual sleep-related deficits as suggested by others.^102^

While residual deficits in the chronic stage might vary and cannot exclude for adaptive network alterations, this approach allowed the assessment of stable alterations less influenced by transient acute effects (such as during hospitalization).

Our study extends the findings of Muñoz-Lopetegi *et al*.,^55^ who investigated sleep from approximately 3 months (Visit 1: median 88 days) up to 1 year after initiation of immunotherapy (Visit 3), at which sleep abnormalities were still common. While prior results highlight the persistence of such sleep-related impairments during the subacute phase, our results clearly indicate a recovery of sleep architecture in LGI1–LE well beyond the subacute stage.

### Sleep-associated spatial memory consolidation

Sleep actively supports the consolidation of spatial memory by coordinating hippocampal reactivation and cortical plasticity.^103-105^ While the DG/CA3 network is known to contribute to spatial encoding, its specific role in sleep-associated consolidation remains less well understood. Previous work from our group showed that sleep stabilizes pattern separation performance in healthy individuals^25^ and that structural damage to the DG predicts pattern separation deficits in LGI1–LE patients.^41^ The current study extends these findings by directly linking behavioural deficits with electrophysiological patterns consistent with the disruption in oscillatory dynamics thought to support systems consolidation during sleep.

Our results reveal that LGI1–LE patients show marked impairments in memory consolidation across sleep, as indicated by a significant reduction in target quadrant dwell time from pre- to post-sleep, compared to stable performance in controls. Importantly, the group difference in post-sleep recall remained significant after controlling for presleep learning performance, indicating that the reduced recall in LGI1–LE patients cannot be explained solely by weaker encoding during the learning phase. These behavioural deficits occurred despite preserved macrostructural sleep and intact EEG power spectra and oscillation densities. Total sleep time, sleep architecture and spectral power in the SO, delta, SWA, theta and spindle bands were comparable across groups. In addition, spindle and SO densities did not differ significantly between patients and controls. While others reported a reduced spindle density in patients with widespread hippocampal damage,^101^ our study specifically targeted DG/CA3 dysfunction in LGI1–LE. The apparently preserved spindle density in our cohort is consistent with the thalamic origin of spindles and suggests that basic memory processing during sleep remains intact.^106-109^ Importantly, spatial task performances did not correlate with subjective sleep quality arguing against sleep disturbances as the main source of behavioural deficits. Although subtle group differences cannot be excluded given the limited sample size, our findings might indicate that the memory impairments are not due to global changes in sleep quantity or spectral power but rather reflect deficits in the functional coordination of sleep-related oscillatory events. Sleep-associated oscillations coordinate hippocampo–neocortical communication, with SOs and spindles mediating interregional transfer and ripples supporting local reactivation.^15,16,110,111^

Critically, our time–frequency analyses suggested that fast spindle activity in patients was not temporally aligned with the SO upstate, in contrast to the clear SO–spindle coupling observed in controls. This descriptive pattern may reflect an impaired local oscillatory coordination during sleep. Although the difference in coupling did not reach statistical significance after cluster-level correction (*P* = 0.1), the observed pattern is consistent with the hypothesis that precise phase alignment—rather than global oscillatory strength (i.e. spindle density or power)—is crucial for memory stabilization. This is congruent with findings by Latchoumane *et al*.,^112^ who showed that only spindles precisely timed to the SO upstate enhanced memory consolidation via SO–spindle–ripple coupling, while mistimed spindles disrupted it.

The behavioural relevance of this potential disruption was further explored by correlation analyses. In LGI1 patients, higher spindle density was associated with poorer place memory performance post-sleep, suggesting that even higher spindle activity does not contribute to memory consolidation when oscillatory coupling is impaired. In contrast, healthy controls showed the hypothesized positive association between spindle density and place memory performance, consistent with sleep’s role in memory consolidation and cognitive flexibility intact.^109^

These findings suggest that DG/CA3 dysfunction in LGI1–LE may disrupt the coordination of hippocampal and cortical oscillatory activity during sleep, thereby impairing memory consolidation. This interpretation is consistent with the visually apparent alteration of phase-specific SO–spindle coupling and by behavioural evidence of post-sleep memory decline.

The observed patterns point to a more fundamental breakdown in the temporal orchestration of systems consolidation—a process critically shaped by coordinated oscillatory dynamics within hippocampal subregions, particularly those centred around DG/CA3.

Within the HC, SWRs, dentate sharp waves (DSWs) and theta–gamma rhythms organize the encoding, reactivation and redistribution of memory traces across cortical sites.^10,24,113-115^ The DG not only gates new information but also functions as a synchronizing hub during sleep-associated consolidation by coordinating DG–CA3–CA1 interactions, thereby supporting synaptic plasticity processes (LTP/LTD) in both hippocampal and cortical circuits.^115-117^

Potential disruption of this coordination—as observed in LGI1–LE—may interfere with the selective stabilization of salient memory traces, as posited by active systems consolidation theory.^10^ Our data are compatible with the possibility that this mechanism may be compromised in LGI1–LE, leading to generalized or unstable memory representations.

These disruptions likely reflect the distinct functional roles that hippocampal subregions play during sleep.^118^ During SWS, the DG shows enhanced activity and drives CA3–CA1 communication via DSWs, especially after spatial learning.^113,114^ This underscores the involvement of DG/CA3 both in encoding and systems consolidation of spatial memory. Disturbances in these mechanisms—as evident in our LGI1–LE patients—may impair the temporal precision and selectivity required for memory stabilization during sleep.

Our findings thus link DG/CA3 dysfunction in LGI1–LE to both physiological (potential oscillatory uncoupling) and behavioural (impaired consolidation) markers of memory disruption. The behavioural deficits and hippocampal atrophy provide robust evidence for disrupted hippocampal-dependent memory processing, whereas the oscillatory findings, including the descriptive SO–spindle coupling pattern and the correlational analyses (inherently exploratory), must be interpreted cautiously and require confirmation in larger samples. Although we lack direct hippocampal recordings, the LGI1–LE model supports a subfield-specific interpretation and underscores the importance of coordinated hippocampal–cortical dynamics for memory consolidation during sleep.

### Shaping of spatial representations during sleep

Pattern separation and pattern completion are well-established hippocampal computations during memory retrieval. It is plausible to assume that similar operations occur during memory reactivation in NonREM sleep, thereby shaping representations at the hippocampal level and, consequently, influencing systems-level consolidation in neocortical networks.^31,115,119,120^ This theoretical framework raises the question of how such dynamics unfold in populations with known hippocampal pathology.

In our LGI1 cohort, spindle density was positively associated with the Place Learning Stability Index, suggesting a disequilibrium not seen in controls. Patients with higher spindle density showed more consistent spatial retrieval in R2 trials with reduced cue configurations. Given known DG dysfunction in LGI1,^41^ this consistency could reflect a shift towards rigid, completion-driven representations, potentially. Instead of enabling adaptive integration, increased spindle activity may have reinforced distorted or overgeneralized spatial patterns.

This hypothesis is further supported by the functional architecture of the DG–CA3 network. The DG supports pattern separation by orthogonalizing similar inputs, while CA3, via recurrent connectivity, enables pattern completion and the integration of shared features into generalized representations.^115,121^ During sleep, these mechanisms may be reengaged as memory traces are reactivated and reorganized. Indeed, CA3 place cells are preferentially reactivated during NREM-associated SWRs, enhancing the accuracy of recent spatial encoding.^117,122,123^

Such processes are shaped by NMDA receptor-dependent plasticity, including asymmetric expansion of place fields, indicating that spatial representations can be modified through experience.^86,124,125^ Building on the prior discussed consolidation mechanisms, delta–ripples and hippocampal spindle oscillations in the DG–CA3 network might facilitate this phenomenon.^115,118,121^ Backward-directed spindles might ‘clear’ the DG network for new inputs, while forward-directed spindles promote long-term potentiation (LTP).^118^ This aligns with the role of CA3 in shaping more generalized representations destined for long-term storage in neocortical networks.

Empirical findings support this dual role of sleep. Hanert *et al*.^25^ found that sleep enhances discrimination between similar spatial inputs in healthy individuals, supporting a role in pattern separation. In contrast, Lutz *et al*.^31,120^ reported that sleep promotes abstraction and reconstructive memory, consistent with pattern completion processes. These findings are not contradictory but reflect different modes of sleep-dependent memory transformation, modulated by task demands and neural integrity.

Our experimental results extend this framework to a clinical population: in contrast to the preserved pattern separation observed in healthy participants,^25^ our LGI1 patients exhibited stability patterns more consistent with completion, in line with Lutz *et al*.,^31^ but possibly driven by dysfunctional attractor dynamics rather than adaptive abstraction. While this interpretation warrants further investigation in larger studies, our present findings are consistent with the hypothesis that sleep-associated spindle activity may promote the transformation of memory content—potentially at the expense of representational specificity in the context of DG–CA3 dysfunction.

### Place learning

In healthy controls, larger hippocampal volumes were associated with more accurate and precise place learning. In contrast, this structure–function relationship was absent in LGI1–LE patients, suggesting a potential disease-specific decoupling of hippocampal integrity and spatial memory performance.

The DG and CA3 subfields—critical for encoding and retrieval of spatial information—are particularly affected in LGI1–LE.^45,52,126^ Mechanistically, LGI1 antibodies disrupt the LGI1–ADAM22/ADAM23 complex, impairing the alignment of presynaptic Kv1 channels with postsynaptic AMPA/NMDA receptors and PSD95.^127-130^ This leads to downregulation of postsynaptic receptor expression and altered excitability of glutamatergic neurons, including place cells primarily located in DG and CA3.^47,126,131^

In line with these observations, our LGI1–LE patients showed significantly reduced hippocampal volumes, particularly in DG and CA3, but also in CA1 and subiculum. While LGI1 expression in CA1 is comparatively lower, atrophy in this region may reflect secondary degeneration due to reduced input from upstream subfields.

Since direct EC–CA1 projections are sufficient for spatial learning in stable environments,^132^ but CA1 damage impairs place learning in humans,^60^ the preserved place learning capacity in our LGI1–LE patients is consistent with intact EC–CA1 connectivity.

Performance deficits became particularly evident during the initial recall phase (R1), which taxed pattern separation. LGI1–LE patients spent less time in the target quadrant and showed a stronger attraction to the nearby lure quadrant, indicating reduced discrimination of highly similar spatial contexts. Such deficits align with the known role of the DG in orthogonalizing overlapping inputs prior to encoding in CA3.^41,89^

In contrast, place memory performance in R2 under partial cues was relatively preserved, particularly in patients with larger CA3 volumes, suggesting that CA3’s recurrent architecture remained at least partially functional. This aligns with rodent studies showing that CA3 rapidly reactivates stored representations from incomplete cues to support memory retrieval in familiar contexts.^133-135^ Supporting this view, Hartley *et al*.^136^ demonstrated that blocking NMDA receptors in CA3 prevents place cell formation, a mechanism critical for spatial memory and navigation.

Accordingly, LGI1–LE patients showed a numerically higher Place Learning Stability Index (R2–R1) compared to controls, suggesting a tendency towards greater reliance on completion-based retrieval processes. This shift may be related to residual CA3 attractor dynamics that may partially compensate for impaired DG input. Distinct remapping properties of hippocampal subfields likely contribute to this dissociation: CA1 place fields adapt flexibly to environmental changes, whereas CA3 fields remain stable across trials.^27,27,137-140^ DG–CA3 communication is essential for encoding distinct spatial patterns, with the DG providing pattern-separated input to the recurrent CA3 network. Reduced DG function may therefore compromise the formation of precise memory traces, while preserved EC–CA1 connectivity supports basic place recognition.^141^ This imbalance may lead to generalized spatial representations and reduced contextual flexibility.

In R3, LGI1–LE patients showed reduced spatial precision and less focused search behaviour compared to controls, suggesting difficulties in retrieving previously learned spatial representations. Supporting this interpretation, experimental studies indicate that both CA3 and the DG are essential for the precision of spatial memory: while CA3 inactivation reduces retrieval accuracy,^142^ DG loss diminishes the selectivity and stability of CA1 place cells.^143^

Consequently, our findings are consistent with the notion that DG–CA3 damage in LGI1–LE impairs the discrimination and precise retrieval of allocentric spatial cues. While place learning and completion-based retrieval remain partially intact—likely via preserved EC–CA1 input and residual CA3 function—the loss of DG-mediated modulation may promote more generalized, less flexible spatial representations, potentially hindering navigation in complex or ambiguous environments.

### Spatial reversal learning

The patients showed marked impairments in spatial reversal learning, requiring them to flexibly adapt to an unexpected change in spatial contingencies. While both groups were confronted with an unexpected change in target location (R4), only healthy controls adapted their search behaviour: they increased their dwell time in the new target quadrant, reduced it in the previously learned location and reached the new target faster and with shorter path lengths. In contrast, LGI1–LE patients failed to adjust their search and showed no improvement in the dependent measures, indicating a lack of flexible updating.

These behavioural findings were further examined using correlation analyses: in controls, better reversal learning performance was associated with higher sleep spindle and SO density, as well as with larger hippocampal and subicular volumes. Importantly, in patients, no such associations were observed. Although correlations do not show causality, our findings are consistent with a potential functional decoupling of structural and oscillatory mechanisms supporting cognitive flexibility.

From a network perspective, these findings may point to a dysfunction of the DG–CA3 network in LGI1–LE. Although dopaminergic and frontostriatal systems also contribute to reversal learning,^144^ our findings underscore the HC—and particularly the DG–CA3 circuit—as critical for encoding altered spatial contingencies and enabling behavioural flexibility.^77,145,146^ This circuit supports engram separation, allowing the distinction between current and outdated reward contingencies by reducing interference from prior learning.^35^ Consistent with this view, hippocampal ensembles have been shown to dynamically reconfigure to encode future goal locations during spatial navigation.^40,147^ Adult-born neurons in the DG may contribute to this process by recruiting distinct CA3 ensembles and thereby enhancing cognitive flexibility.^145,146^

In our healthy controls, these mechanisms likely enabled the formation of updated spatial representations and suppression of obsolete ones. In contrast, patients’ inability to disengage from the previously rewarded location suggests reduced remapping capacity, possibly due to impaired pattern separation. This may reflect a breakdown in how the hippocampal system updates the meaning and salience of spatial cues, which normally allows downstream CA1–subiculum circuits to distinguish between competing sequences and reward contingencies.^144^

Experimental studies support this interpretation: animals with hippocampal lesions show reduced flexibility in adapting to novel environments,^148^ whereas intact hippocampal circuits enable efficient remapping and suppression of obsolete behaviour.^24,149,150^ This flexibility is thought to depend on dentate-driven ripple activity that enables prospective firing in CA3 neurons,^24^ thereby supporting anticipatory memory encoding—a mechanism—that may be disrupted in our LGI1–LE patients.

Thus, our findings suggest that impaired DG–CA3 function in LGI1–LE limits the flexible adjustment of spatial behaviour in response to altered reward contingencies and may, in part, contribute to behavioural rigidity in dynamic environments.

### Limitations

The small sample size, which is largely determined by the rarity of LGI1–LE, represents a potential limitation as it may restrict the generalizability of the findings. A *post hoc* sensitivity analysis (G*Power 3.1) indicated that with *n* = 15 per group, the present design provided 80% power to detect small-to-medium interaction effects (*f* = 0.24, ηp^2^ ≈ 0.05). The main findings, including the significant group × time effect for sleep-associated consolidation (*f* = 0.43, ηp^2^ = 0.157), the robust group effects during initial place learning (B1–B2; *f* ≈ 0.60–0.67) and the medium-to-large interaction effects during reversal learning (R3–R4; *f* ≈ 0.45–0.49), all exceeded this sensitivity threshold. This suggests that the core results were adequately powered despite the limited cohort size. While this *post hoc* approach cannot replace an *a priori* power calculation, it provides an estimate of the sensitivity and interpretive limits of the present sample. However, smaller or more subtle group differences may still have remained undetected and should therefore be interpreted with caution, limiting the confidence in observed null findings.

Additionally, the experimental design, which focused on variable spatial arrangements to assess allocentric learning and to capture pattern separation and completion, may also represent a limiting factor. Patients showed mild reductions in executive and working-memory performance (TMT, Digit Span), yet these abilities were not related to allocentric spatial navigation. The present task primarily engaged hippocampal-dependent memory retrieval rather than strategic planning or attentional control, and both groups performed under identical instructions and environmental conditions, making systematic strategy differences unlikely. While individual differences in navigation strategy cannot be excluded, strategy use was not formally assessed and is noted as a limitation.

Although the conditions on Day 2 were presented in immediate succession, the task context and goal remained constant, reducing the likelihood of meaningful interference between conditions. All participants completed the conditions in the same fixed order, ensuring that any residual carry-over would have affected both groups equally. No systematic performance drift was observed across the sequence of recall conditions, further arguing against cross-condition interference.

An advantage of focusing on the chronic phase of LGI1–LE patients is the ability to observe the long-term residual effects of the disease. This approach allows the assessment of stable behavioural and structural alterations that are less influenced by transient effects of the acute disease stage. However, it also introduces a potential drawback: residual conditions vary within the cohort, and compensatory mechanisms—such as adaptive network reorganization following immunotherapy and partial recovery—may contribute to the observed patterns. Although we carefully matched the cohorts, potential age-related effects may still impact the patient cohort, possibly amplifying the role of such compensatory mechanisms. To further elucidate these findings, subsequent studies are necessary.

## Conclusion

This study highlights the critical role of the DG–CA3 circuit in spatial memory processing, sleep-associated consolidation and cognitive flexibility.

Despite normalized sleep architecture in the chronic phase of LGI1-associated LE, patients showed persistent impairments in HC-dependent memory functions. Specifically, they exhibited deficits in spatial pattern separation, imprecise place learning and pronounced impairments in spatial reversal learning. These deficits were associated with structural DG–CA3 damage and a potential functional decoupling of sleep-related oscillatory mechanisms, particularly with respect to the SO–spindle coupling. While basic sleep features were preserved, altered spindle dynamics may have reinforced distorted or rigid spatial representations rather than supported adaptive consolidation.

Our findings suggest that DG–CA3 dysfunction in LGI1–LE disrupts the formation and updating of precise spatial representations, leading to inflexible memory retrieval and reduced adaptability in dynamic environments. These results underscore the subfield-specific vulnerability of hippocampal computations and their relevance for sleep-dependent systems consolidation in clinical populations.

## Supplementary Material

fcag255_Supplementary_Data

## Data Availability

The data presented in this work are available upon reasonable request. Analysis code (including scripts used for the time–frequency and coupling analyses) is publicly available at https://github.com/yf-neuro/LGI1-coupling-analysis.git.
