# Supplementary material for: Impaired consolidation of spatial memory during sleep in patients with leucine-rich glioma-inactivated 1-associated limbic encephalitis
Source: Brain Commun. 2026 Jul 6;8(4):fcag255. doi: 10.1093/braincomms/fcag255 (PMC13373790; doi:10.1093/braincomms/fcag255)
Supplement: fcag255_Supplementary_Data [file fcag255_supplementary_data.docx]

**Supplementary Materials**

***
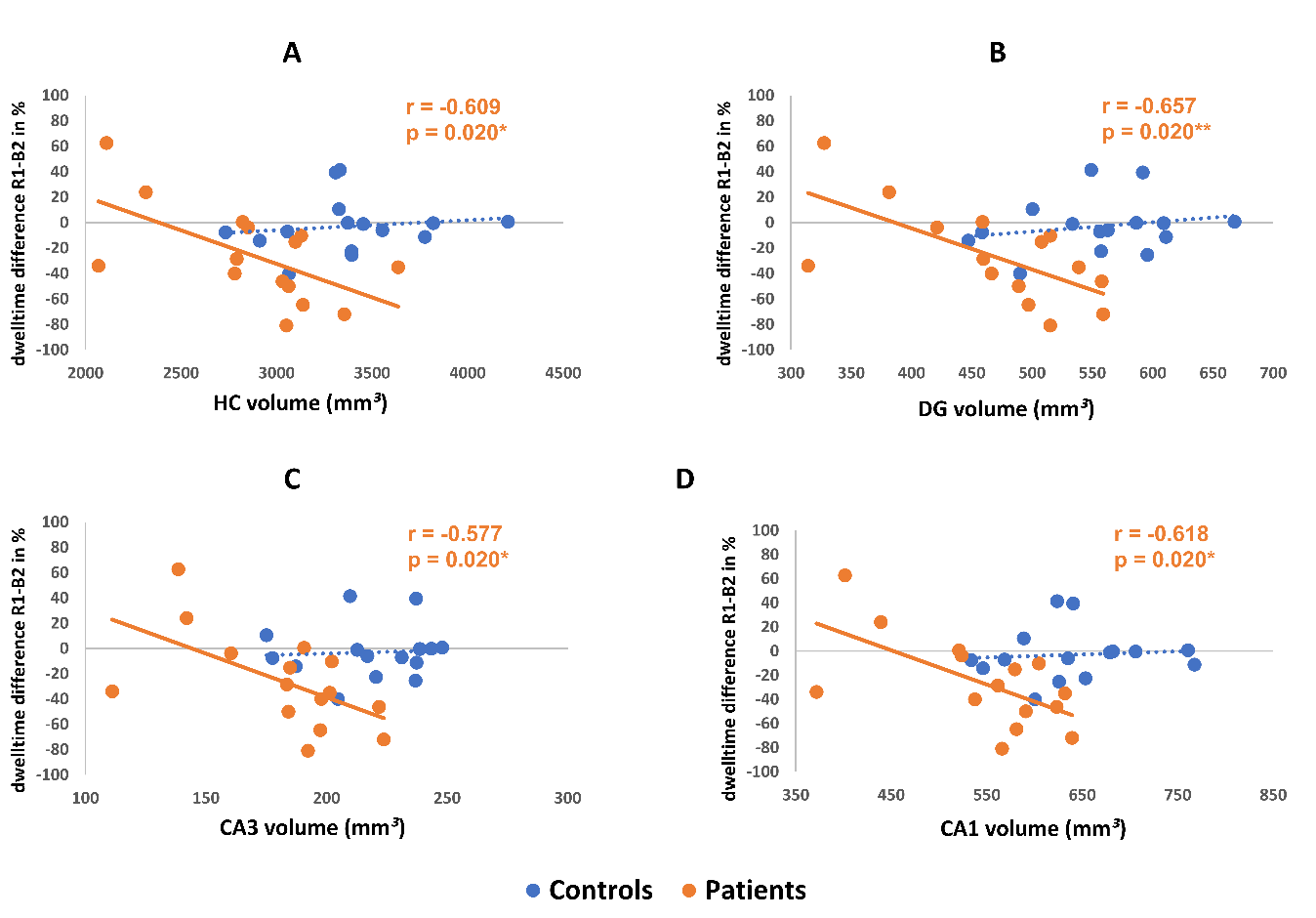
***

***Supplementary Figure S1:*** ***Correlations between hippocampal volumes and spatial memory consolidation*** *(Difference in relative target quadrant dwell time, R1-B2). The results for both patients and controls are plotted.* ***A****: total HC volume,* ***B****: DG volume,* ***C****: CA3 volume,* ***D****: CA1 volume correlations are shown. Each data point represents one individual participant (orange = LGI1-LE patient, blue = healthy control).*  *Bold lines indicate statistically significant correlations; dotted lines are for visualization only. Pearson correlation analyses were performed. N = 15 LGI1-LE patients, 15 healthy controls. P-values were corrected for 5 comparisons (Subiculum not shown); * p <0.05.*


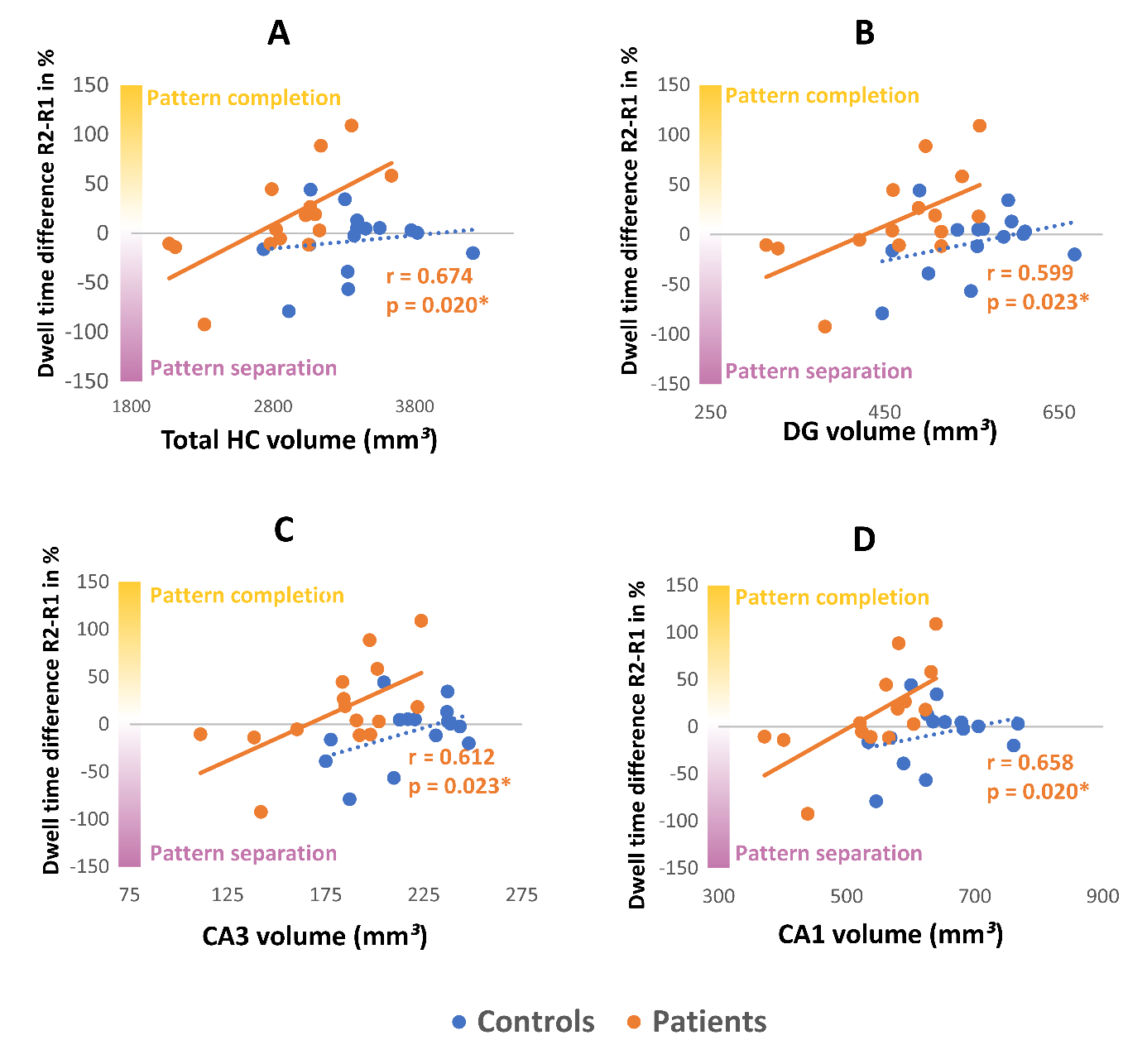


***Supplementary Figure S2:*** ***Correlations between hippocampal subfields and the Place Learning Stability Index*** *(Dwell time R2-R1). Shown are correlations between the difference in the relative dwell time (learned target quadrant) in R1 and R2 (calc. R2 - R1) with* ***A****: Total HC,* ***B****: DG,* ***C****: CA3,* ***D****: CA1. Each data point represents one individual participant (orange = LGI1-LE patient, blue = healthy control).* *The yellow / purple* *column marks the spectrum from pattern separation in R1 to pattern completion in R2 as a further visual reference for the stability marker R2-R1. Please note that patients' performance is relatively shifted towards pattern completion, resulting in a functional disequilibrium. Bold regression lines indicate significant correlations; dotted lines are for visualization only. Pearson correlation analyses were performed. N = 15 LGI1-LE patients and 15 healthy controls. P-values were corrected for 5 comparisons (Subiculum not shown); * p <0.05.*
